# Neuroprotective Effects of Olive Oil: A Comprehensive Review of Antioxidant Properties

**DOI:** 10.3390/antiox13070762

**Published:** 2024-06-24

**Authors:** Marta Gonçalves, Nuno Vale, Paula Silva

**Affiliations:** 1 Laboratory of Histology and Embryology, Department of Microscopy, School of Medicine and Biomedical Sciences (ICBAS), University of Porto (U.Porto), Rua Jorge Viterbo Ferreira 228, 4050-313 Porto, Portugal; martamariacg2002@gmail.com; 2 PerMed Research Group, Center for Health Technology and Services Research (CINTESIS), Rua Doutor Plácido da Costa, 4200-450 Porto, Portugal; nunovale@med.up.pt; 3 CINTESIS@RISE, Faculty of Medicine, University of Porto, Alameda Professor Hernâni Monteiro, 4200-319 Porto, Portugal; 4 Department of Community Medicine, Information and Health Decision Sciences (MEDCIDS), Faculty of Medicine, University of Porto, Rua Doutor Plácido da Costa, 4200-450 Porto, Portugal; 5 iNOVA Media Lab, ICNOVA-NOVA Institute of Communication, NOVA School of Social Sciences and Humanities, Universidade NOVA de Lisboa, 1069-061 Lisbon, Portugal

**Keywords:** olive oil, neurodegenerative diseases, neuroprotection, olive oil polyphenols, antioxidants

## Abstract

Neurodegenerative diseases are a significant challenge to global healthcare, and oxidative stress plays a crucial role in their development. This paper presents a comprehensive analysis of the neuroprotective potential of olive oil, with a primary focus on its antioxidant properties. The chemical composition of olive oil, including key antioxidants, such as oleuropein, hydroxytyrosol, and oleocanthal, is systematically examined. The mechanisms by which these compounds provide neuroprotection, including counteracting oxidative damage and modulating neuroprotective pathways, are explored. The neuroprotective efficacy of olive oil is evaluated by synthesizing findings from various sources, including in vitro studies, animal models, and clinical trials. The integration of olive oil into dietary patterns, particularly its role in the Mediterranean diet, and its broader implications in neurodegenerative disease prevention are also discussed. The challenges in translating preclinical findings to clinical applications are acknowledged and future research directions are proposed to better understand the potential of olive oil in mitigating the risk of neurodegenerative conditions. This review highlights olive oil not only as a dietary component, but also as a promising candidate in preventive neurology, advocating for further investigation in the context of neurodegenerative diseases.

## 1. Introduction

Neurodegenerative diseases are a diverse set of disorders that involve the progressive degeneration of the central or peripheral nervous system. These diseases are characterized by a gradual loss of neurons, leading to impairments in motor and cognitive functions [1,2]. Examples of neurodegenerative diseases include Alzheimer’s disease (AD), Parkinson’s disease (PD), Huntington’s disease (HD), and amyotrophic lateral sclerosis (ALS). Notably, these diseases are not limited to neuronal cells, but also involve metabolic dysfunction in the brain, spinal cord, and nerves [2]. The molecular mechanisms underlying these diseases remain largely unknown, and researchers are actively seeking effective biomarkers for early diagnosis and therapeutic interventions [3]. The complexity of neurodegenerative diseases is further compounded by the involvement of miRNAs in post-transcriptional gene regulation, suggesting a critical epigenetic control mechanism that could play a role in the development and potential treatment of these conditions [4]. 

The epidemiology of neurodegenerative diseases is characterized by a significant global health burden, with an increasing prevalence attributed to aging populations and extended life spans [5]. AD and PD are among the most extensively studied in terms of epidemiological data, with environmental factors such as mental and physical activity, diet, and neurotoxin exposure being implicated in their incidence [6]. The Global Bur of Disease 2019 study provides comprehensive estimates indicating that neurological disorders, including neurodegenerative diseases, are responsible for nearly 10 million deaths and 349 million disability-adjusted life years (DALYs) globally, with AD and PD showing a marked increase in DALYs from 1990 to 2019 [7]. Interestingly, while the overall burden of neurological disorders has increased, there is significant variation across regions, suggesting the need for region-specific strategies [7]. Additionally, the recent COVID-19 pandemic has been associated with both the modification of existing neurological disorders and the potential for future neurodegenerative diseases, highlighting the complexity of epidemiological patterns [8]. Currently, the treatment options focus on the symptoms rather than on preventing the loss of neurons [9].

Neuroprotection is of paramount importance, as it encompasses strategies aimed at preserving neuronal structure and function, thereby preventing neuronal cell death, which is a common endpoint in various neurological disorders [10]. The significance of neuroprotection is underscored by the fact that neurological disorders, including neurodegenerative diseases and acute injuries, lead to progressive neuronal dysfunction and loss, contributing to morbidity and mortality worldwide [11]. Currently, there are several potential neuroprotective agents, and their respective mechanisms of action have been identified. These include compounds such as coenzyme Q10 [12,13] and polyphenols with antioxidative and anti-inflammatory properties [14,15,16]. Additionally, the neuroprotective potential of neuroEPO, which is a modified form of erythropoietin that has been engineered to have neuroprotective effects without significantly stimulating erythropoiesis, in various neurological disorders has been highlighted, with observed benefits in ischemic or degenerative brain damage [17,18].

## 2. Neurodegenerative Diseases and Oxidative Stress

Oxidative stress is crucial in the pathophysiology of neurodegenerative diseases. It serves as both a symptom and a potential cause of neuronal decline [19]. Owing to their high metabolic activity, neurons require a significant amount of oxygen, which leads to the production of reactive oxygen species (ROS) and other free radicals [19]. The presence of polyunsaturated fatty acids in neuronal membranes renders them particularly susceptible to oxidative stress [20,21]. Additionally, the brain’s high iron (Fe) content can catalyze the formation of more damaging free radicals. The relative deficiency of antioxidant defenses, such as superoxide dismutase (SOD) and glutathione peroxidase (GPx), further exacerbates this vulnerability [21]. Neurodegenerative disorders, including AD [22,23], PD [23,24], HD [23,25], and ALS [23,26], exhibit elevated levels of oxidative stress markers not only in the brain but also in peripheral tissues. This systemic manifestation of oxidative stress has a broad effect on neuronal integrity. The origin of oxidative stress in these conditions is multifaceted and is influenced by environmental factors, genetic predispositions, and their interactions, resulting in widespread damage to lipids, proteins, and DNA within neural tissues [23]. Disturbances in the balance of metals such as Fe and copper are also crucial in the development of neurodegenerative diseases [23]. These metals can catalyze the formation of highly reactive hydroxyl radicals (HO^•^) through the Fenton reaction, thereby intensifying oxidative stress and cellular damage [23]. The role of oxidative stress extends to the specific mechanisms involved in a diverse range of neurodegenerative diseases.

**Figure 1:**
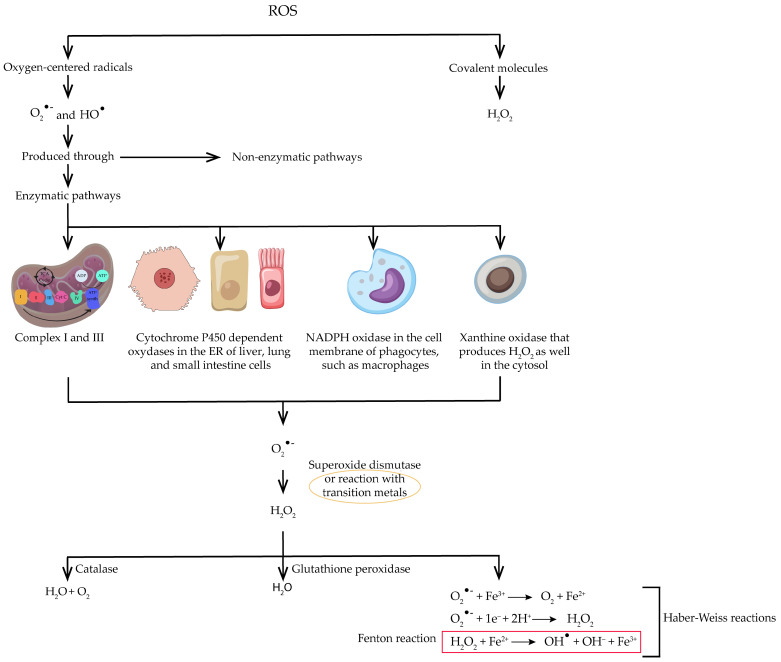
Generation and metabolism of reactive oxygen species (ROS). ROS are produced during cell metabolism and play crucial roles in normal cell function. However, they can be harmful when present in excess. ROS can be categorized into two groups: oxygen-centered radicals, such as superoxide anions (O_2_^•−^) and hydroxyl radicals (HO^•^), and covalent compounds, such as hydrogen peroxide (H_2_O_2_). O_2_^•−^ is produced in various cellular compartments, including mitochondria, where O_2_^•^ − is a byproduct of the electron transport chain; endoplasmic reticulum (ER), in the liver, lung, and small intestine cells, cytochrome P450-dependent oxygenase generate O_2_^• −^; in cell membranes of phagocytes, NADPH oxidase (NOX) catalyzes the production of O_2_^• −^; in cytosol, xanthine oxidase produces H_2_O_2_. O_2_^•−^ can be converted to H_2_O_2_ by superoxide dismutase (SOD) or through reactions with transition metals. The Haber–Weiss reactions, including the Fenton reaction, are noteworthy because they lead to the formation of HO^•^ from H_2_O_2_. Endogenous antioxidant mechanisms, such as glutathione peroxidase (GPx), reduce H_2_O_2_ to water, whereas catalase transforms it into oxygen and water.

Free radicals, which include ROS and reactive nitrogen species (RNS), are generated by cell metabolism and are fundamental in normal cell function, while potentially damaging when in higher concentrations. In fact, as depicted in Figure 1, ROS can be subdivided into oxygen-centered radicals, such as superoxide anion (O_2_^•−^) and HO^•^ or covalent molecules, for instance hydrogen peroxide (H_2_O_2_). While the former do not appear to be nearly as reactive as H_2_O_2_, O_2_^•−^ is the precursor of several ROS. There are several sites to produce this anion, including the mitochondria, as a result of the electron transport chain; the endoplasmic reticulum (ER) of liver, lung, and small intestine cells, where cytochrome P450-dependent oxygenases produce it; the cell membrane of phagocytes through the action of nicotinamide adenine dinucleotide phosphate (NADPH) oxidase (NOX); and the cytosol, via the action of the enzyme xanthine oxidase that produces H_2_O_2_ as well. Moreover, in a pathway that does not involve enzymes, reduced coenzymes, prosthetic groups, and reduced xenobiotics can transfer a single electron to oxygen, therefore originating O_2_^•−^. This anion can undergo several reactions, including conversion into H_2_O_2_ by SOD or through reaction with transition metals. In this sense, the Haber–Weiss reactions, which include the Fenton reaction, appear to have significant importance, as they allow for the production of HO^•^ from H_2_O_2_. Furthermore, GPx can reduce H_2_O_2_ to water and catalase can transform it into oxygen and water, meaning they constitute endogenous antioxidant mechanisms [27,28].

As organisms age, their once relatively high resistance to exogenous factors that may cause oxidative stress decreases, causing ROS/RNS-mediated late-onset diseases, which include neurodegenerative diseases [28]. The brain is an organ that consumes about 20% of total basal oxygen and is susceptible to oxidative stress due to several factors, depicted in Table 1 [29].

The neurodegenerative process shares certain characteristics with aging, such as lipofuscin accumulation and neurofibrillary degeneration. Neurofibrillary degeneration is a consequence of the formation of intracellular neurofibrillary tangles, which are commonly found in AD and are composed of phosphorylated high molecular weight neurofilament proteins, microtubule-associated protein 2, microtubule-associated protein tau (often hyper-phosphorylated), and ubiquitin. Research has suggested that ROS plays a role in tau phosphorylation. Additionally, oxidative stress leads to the activation of c-Jun N-terminal kinases and p38 and inactivation of protein phosphatase 2A, which contributes to the phosphorylation of tau. Furthermore, phosphorylated tau is more susceptible to oxidative stress-induced modifications, resulting in its misfolding and aggregation. In contrast, extracellular amyloid deposition in the gray matter is another hallmark of AD [28]. The current understanding of AD pathogenesis emphasizes the cleavage of amyloid precursor protein (APP) into the Aβ peptide, which subsequently aggregates into plaques. Some studies suggest that the toxicity associated with these plaques may contribute to the morphological changes observed in the tau protein [30]. The amyloidogenic pathway of APP is believed to be triggered by reactive species, which also promote the elevated activity of β-secretase. Consequently, this results in the accumulation of Aβ peptide [28]. Table 2 presents the effects of ROS on mechanisms related to the development of AD, which were reviewed elsewhere.

PD symptoms can be subdivided into motor and non-motor symptoms, including depression, sleep disorders, and cognitive deficits [31]. Motor symptoms are primarily caused by a reduction in neurons in the substantia nigra pars compacta and loss of dopamine content. Additionally, the presence of Lewis bodies is a significant characteristic of neurodegenerative diseases. These structures are eosinophilic inclusions with a dense core and a pale-stained halo of radiating filaments that contain misfolded α-synuclein (α-Syn). The neuropathological presentation of PD extends beyond the dopaminergic nuclei and involves non-dopaminergic areas. The presence of Lewis bodies in these areas is the reason for the non-motor symptoms of this pathology. It is essential to note that the pathology of PD does not originate in the substantia nigra pars compacta, but rather in the olfactory bulb and lower brain stem before progressing to the midbrain and eventually extending to cortical regions [32]. Oxidative stress can induce the oligomerization of α-Syn (Table 2) [28]. Dopamine is typically transported into synaptic vesicles by the vesicular monoamine transporter. However, in damaged neurons, there may be excess dopamine outside the vesicles, which can be metabolized by either monoamine oxidase (MAO) or through auto-oxidation via ROS. This can lead to dopamine-mediated toxicity and oxidative stress, resulting in mitochondrial dysfunction and alterations in several fundamental proteins in PD, including α-Syn. The dopamine quinones resulting from auto-oxidation can be further oxidized to form aminochrome, which can in turn result in the formation of a superoxide radical and a decrease in NADPH [31]. It is essential to emphasize that the detrimental metabolism of dopamine is likely attributable to the production of 3,4-dihidroxyphenylacetaldehyde (3,4-DHPAA), which plays a crucial role in potentiating the loss of dopaminergic neurons in PD [33].

The etiology of HD is marked by the expansion of a trinucleotide repeat in the CAG sequence of the Huntington protein, resulting in an abnormally lengthy protein [34]. There is a significant probability that the mutant Huntington protein aggregates, which may result in neurotoxicity. Notably, oxidative stress can exacerbate the aggregation of this protein, ultimately leading to cell death [28]. Accumulation of this complex impairs the mitochondrial respiratory chain, resulting in ROS production. Decreased mitochondrial function contributes to disease progression (Table 2) [23].

ALS is a progressive neurodegenerative disease with an unknown etiology that is characterized by rapid degeneration of motor neurons in both the upper and lower nervous systems. The pathogenesis of ALS is multifactorial, involving genetic predisposition, glutamate excitotoxicity, oxidative stress, mitochondrial dysfunction, and other factors; however, it is not fully understood. Symptoms of ALS include muscle weakness, atrophy, and paralysis, which typically lead to death within 3–5 years of diagnosis. Interestingly, although the etiology of ALS is still unknown, certain genetic and environmental factors have been identified as contributing to its onset and progression [35]. For instance, familial ALS accounts for 5–10% of cases, indicating a genetic component, whereas sporadic ALS suggests other etiological factors. Additionally, the ALS split-hand phenomenon, where there is selective atrophy of the hand muscles, points to differential cortical representation as a possible factor in the manifestation of the disease [36]. Table 2 presents crucial information regarding the various components that contribute to ROS generation and accumulation in ALS. Mitochondrial dysfunction caused by gene mutations results in impaired mitochondrial function, reduced ATP production, and increased ROS production. This table also emphasizes the impact of metal ion imbalances, particularly elevated Fe levels, which exacerbate oxidative stress through the Fenton reaction. Inflammation, which is driven by the activation of astrocytes and microglia, contributes to oxidative stress through the production of pro-inflammatory cytokines and activation of NADPH oxidase. The decline in natural antioxidant defenses, including a reduction in key enzymes, such as GPx and catalase, is a significant factor in the accumulation of ROS. Genetic factors such as mutations in SOD1 result in the formation of misfolded proteins that exacerbate oxidative stress and mitochondrial dysfunction. Environmental factors, including exposure to pesticides, metals, and electromagnetic fields have also been identified as contributors to oxidative stress and neuronal damage. Moreover, the accumulation of misfolded protein aggregates in motor neurons has been described as a cause of oxidative and inflammatory damage, perpetuating a cycle of oxidative stress and neuroinflammation. Finally, overactivation of glutamate receptors, particularly N-Methyl-D-Aspartate (NMDA) receptors, leads to excessive Ca^2+^ influx into neurons. The resulting increase in intracellular Ca^2+^ levels disrupt the mitochondrial function, leading to ROS generation. This cascade of events exacerbates OS and contributes to motor neuron degeneration. Hence, this dysfunction in Ca^2+^ homeostasis and mitochondrial impairment results in sustained OS, which plays a crucial role in ALS pathophysiology [37].

**Table 2:** Reactive oxygen species origin in neurodegenerative disorders.

	AD	PD	HD	ALS
Mitochondrial dysfunction	Deficiency in cytochrome C oxidase → impaired normal flow of electrons → increased electron leakage and ROS production. Mutations in mitochondrial DNA → impaired ETC components → increased ROS generation, and changes in gene expression affecting mitochondrial function further exacerbate this process [22,23,38].	Accumulation of misfolded α-Syn protein → inhibits complex I of the ETC → disrupted electron flow → increased ROS production. Mitochondrial dysfunction → microglia activated → production of inflammatory cytokines and additional ROS → damaged neurons and mitochondria → OS. Elevated intracellular Ca^2+^ levels → activation of mitochondrial enzymes and disruption of the mitochondrial membrane potential → ROS production and decreased ATP synthesis. Mutations in *PINK1* and *Parkin* genes → decreased mitochondrial quality control → complex I deficits → increased ROS production. Increased fragmentation and altered trafficking of mitochondria → impaired function of the organelle and increased ROS production [23,39,40,41,42].	mHTT → impaired mitochondrial function → inefficient ATP production and increased electron leakage → leaked electrons interact with molecular oxygen → ROS formation → further impairment of mitochondrial function → neuronal damage and cell death. Mutations in the mitochondrial D-loop of mitochondrial DNA → defective respiratory chain complex activities → mitochondrial dysfunction → increased ROS production. Elevated levels of OS precede HD symptoms, indicating that OS plays a critical role in HD pathogenesis. mHTT → increased sensitivity of the mitochondria to Ca^2+^ induced permeability transition → mitochondrial swelling and depolarization → ROS generation → neuronal cell death [23,43,44].	Mutations in *SOD1*, *TDP-43*, and *C9orf72* genes (commonly linked to familial ALS) → impaired mitochondrial respiratory chain → decreased ATP production and increased electron leakage → ROS production → further impairment of mitochondrial function. Impaired mitochondrial function → excessive Ca^2+^ levels → stress the mitochondria → increased ROS production and mitochondrial permeability transition → neuronal damage [23].
Metal ion imbalance	Fe accumulation → ROS production through Fenton reactions when homeostasis is disrupted → OS. Post-mortem studies: Accumulation of Fe^3+^ → senile plaque and neurofibrillary tangle formation. Increased Fe load and toxicity → regulated cell death mechanism caused by the accumulation of lipid peroxidation products (ferroptosis). Free Cu accumulation → ROS production through Fenton reactions. Cu binds to Aβ plaques → increased aggregation and toxicity of Aβ and generation of ROS. •APP contains a Cu-binding site, which, when occupied by Cu, promotes oxidative modification of the protein. Zn competes with Cu and Fe for binding sites and a disturbance in its regulation can affect the balance of these metals. Zn accumulation in amyloid plaques → disrupted metal homeostasis → increased OS. Zn → influences the activity of various enzymes involved in the OS response [45,46].	Accumulation of Fe (linked to NM, a dark pigment that accumulates in dopaminergic neurons and is synthesized from oxidized DA) → increased free radical production, OS, and neurodegeneration. Dopaminergic neurons, rich in Fe, are highly susceptible to Fenton’s or Haber–Weiss reactions. Fe → facilitates aggregation of formation of α-Syn → formation of Lewy bodies. Fe-dependent binding of α-Syn to cyto-chrome c and mitochondrial damage → increased susceptibility of the substantia nigra to free radicals [47].	Excessive Fe in the brain → increased OS → neurodegenerative processes. mHTT → disrupted normal Fe homeostasis → Fe accumulation in the neurons → ROS production → oxidative damage and mitochondrial dysfunction. Impaired mitochondrial function, in turn, exacerbates Fe-induced OS, creating a vicious cycle of neuronal damage and cell death [23,42].	Elevated levels of Fe have been observed in various regions of the brain, particularly in the motor cortex and spinal cord. Mutations in SOD1 → accumulation of Fe → ROS production via the Fenton reaction. Mutations in SOD1 can lead to the improper handling of Fe and other metals, further promoting OS. The Fe storage proteins ferritin-H and ferritin-L are increased in ALS patients, indicating an attempt by cells to sequester excess Fe and mitigate its toxic effects, but this mechanism often fails to prevent the Fe-induced oxidative damage. Fe → dysfunction of mitochondrial enzymes that contain Fe–sulfur clusters → impaired electron transport and increased leakage of electrons → formation of ROS [48].
Inflammation	Aβ plaque buildup → activation of microglia and astrocytes → ROS production and production of pro-inflammatory cytokines (TNF-α, IL-1β, and IL-6) → cytokines cross the BBB drive neuroinflammation and increase Aβ and tau protein levels, while reducing antioxidant enzyme expression. Prolonged immune activation results in low-grade chronic inflammation, known as inflammaging, which is marked by elevated systemic cytokine levels even without infection. Chronic inflammation → damaged BBB → peripheral immune cells enter the brain → inflammation. Chronic inflammation → activated NOX → ROS production. Chronic inflammation → impaired mitochondrial function → ROS production MetS (common in patients with AD) → intensifies ROS production, damages blood vessels, disrupts BBB integrity and Aβ clearance. AGEs interaction with their receptors during chronic inflammation → higher ROS levels. Chronic stress → activated HPA axis → heightened glucocorticoid levels → increased Aβ deposition and neuronal damage while priming microglia for an excessive inflammatory response → ROS production [49,50,51,52,53,54,55,56].	Microglia activation → production of pro-inflammatory cytokines and chemokines → activation of the NF-κB pathway → ROS generation. Activated microglia generate ROS through enzymes, such as NOX. Misfolded proteins and environmental toxins → activation of the NLRP3 inflammasome → production of IL-1β and IL-18 → inflammation and ROS production → chronicity of neuroinflammation in PD. Neuroinflammation → impaired mitochondrial function → increased ROS production. Chronic neuroinflammation → accumulation of misfolded proteins such as α-Syn → further activation of microglia and perpetuation of the inflammation cycle and ROS production. Chronic neuroinflammation → decreased antioxidant defenses including GSH [42,57,58,59].	Elevated levels of pro-inflammatory cytokines and decreased levels of anti-inflammatory cytokines are correlated with disease progression. Pro-inflammatory stimuli → activated microglia → release of IL-1β, IL-6, and TNF-α → production of ROS. Studies have found a significant accumulation of activated microglia in the striatum and frontal cortex of HD brains, with the density of activated microglia correlating with the severity of pathology. Mitochondrial dysfunction in microglial cells → suppressed IL-4-induced alternative response, responsible for attenuation of inflammation → increased expression of pro-inflammatory mediators → neuronal death [23].	Activation of astrocytes and microglia leads to neuroinflammation → increased OS. Activated glial cells → production of pro-inflammatory cytokines and other inflammatory mediators → generation of ROS, causing oxidative damage to neuronal cells and exacerbating the progression of neurodegeneration. Neuroinflammation → increased activity of NADPH oxidase (enzyme responsible for producing O_2_^•–^, a type of ROS). In familial ALS, mutations in SOD1 have been found to enhance the pro-oxidant activity of this enzyme, further increasing ROS production, and contributing to motor neuron damage [23,37,60].
Reduced antioxidant defenses	Decreased brain antioxidant systems, such as SOD, GPx, and catalase → failure in counterbalancing the increase in ROS production. SOD levels can be induced or reduced by OS, with various studies showing differing results. Similar to SOD, GPx levels may vary under OS conditions. Studies also indicate a significant decrease in catalase activity in specific brain regions of patients with AD, such as the parietotemporal cortex, basal ganglia, and amygdala, which aligns with the observed decline in antioxidant defenses in AD [61,62,63].	Decreased GSH levels (reduction in the ratio of GSH to GSSG) → OS and mitochondrial dysfunction → neurodegeneration. High levels of ROS and RNS → impaired function of mitochondrial complex I and decreased the activity of GSH reductase (enzyme responsible for reducing GSSG to GSH) [64].	Decreased activity of antioxidants, such as SOD and ascorbate → increased oxidative damage. Studies on human HD patients have identified increased markers of oxidative damage in brain tissues, including cytoplasmic lipofuscin, DNA strand breaks, and oxidative markers in DNA bases. Other cellular macromolecules accumulate oxidative damage, associated with protein nitration and lipid peroxidation. HD patients also exhibit increased levels of oxidative damage markers, such as 8-hydroxydeoxyguanosine and MDA, decreased activities of antioxidant enzymes Cu/Zn-SOD and GPx in erythrocytes, and reduced catalase activity in skin fibroblasts [65].	ALS is characterized by a decline in the natural antioxidant defenses of the neurons. This includes reductions in enzymes such as GPx and catalase, which normally help detoxify ROS [66].
Genetic factors	ApoE protein, which exists in several isoforms, plays a crucial role in lipid metabolism, Aβ deposition, and OS. ApoE4 has the lower antioxidant capacity compared to ApoE2 and ApoE3, due to its reduced ability to bind and neutralize lipid peroxidation products such as HNE → presence of the ApoE4 allele is associated with increased OS. ApoE4 is a significant genetic risk factor for AD. Mitochondrial dysfunction, which is influenced by genetic factors → increased ROS production. Reduced activity of SIRT3 → increased ROS production → neurodegeneration [67,68,69,70].	Mutations in the α-Syn gene → formation of protofibrils → generation of pores in cellular membranes and disruption in cellular homeostasis. Protofibrils → increased cytoplasmic DA concentrations → rapid oxidation and production of DAQs, O_2_^•–^, and H_2_O_2_. Mutations in parkin gene → impaired mitochondrial function and an increased sensitivity to mitochondrial toxins → higher accumulation of OS markers and mitochondrial dysfunction. Mutations in DJ-1 (encoded by PARK7) → reduced antioxidative activity, due to decreased GSH → increased OS [71,72,73].	Mutation in HTT → impaired mitochondrial function → ROS formation → neuronal damage and cell death. Mutations in D-loop of mitochondrial DNA → mitochondrial dysfunction → heightened ROS production [23,43].	Mutations in the SOD1 gene → formation of misfolded and aggregated SOD1 proteins → loss of normal antioxidant function and acquirement of pro-oxidant properties → increased OS. Misfolded SOD1 protein → stimulate NADPH oxidase → ROS overproduction. Mutated SOD1 proteins → mitochondrial dysfunction → ROS production [26].
Environmental factors	Deficiencies in essential nutrients such as folate and Fe can lead to OS. Studies have shown that folate deprivation combined with excess dietary Fe increases oxidative damage in the brain, which is not fully mitigated by elevated GSH levels. Environmental exposure → chronic inflammation → overproduction of free radicals and OS. Dysbiosis or an imbalance in the gut microbiome → increased LPS production → triggered OS and inflammation in the brain. Exposure to environmental toxins such as heavy metals → production of ROS → cellular and mitochondrial damage [67,68,69,70].	Exposure to environmental toxins, particularly heavy metals, has been recognized as a significant risk factor for PD. Mn exposure → neurotoxic accumulation in the brain Fe → oxidative damage through the Fenton reaction Hg exposure → ROS production and impaired detoxifying proteins. Pb exposure → disruption of neurotransmitter systems and induction of oxidative and mitochondrial stress → dopaminergic neuronal death. [42,74].	The relationship between HD and environmental factors needs to be further assessed, as no specific and clear correlations have been established [75].	Pesticides (organophosphates) → lack of function of cholinesterase → excessive accumulation of acetylcholine in the central nervous system → depleted mitochondrial ATP synthesis → ROS overproduction → oxidative damage and neurodegeneration. The metal Al has pro-oxidant properties and the ability to weaken antioxidant defenses, having a role in disease progression. Mn crosses the blood–brain barrier → impaired ATP production → pro-oxidative state → motor neuron damage. Exposure to EMFs → generation of free radicals and reactive species → motor neuron death [76].
Protein aggregation and accumulation	Aβ accumulates primarily in mitochondria → disruption of normal oxidative phosphorylation → increased electron leakage → ROS production. Aβ peptides undergo a process known as “radicalization,” where they become sources of free radicals, intensifying OS within neurons and contributing to neuronal damage and death. Aβ aggregates → activation of microglia and resident immune cells of the brain → production of inflammatory mediators (NO) → reaction with ROS → formation of highly reactive peroxynitrite (compound that can nitrate tyrosine residues in proteins, thereby altering their function) [77].	α-Syn → ferrireductase activity (reduction of Fe^3+^ to Fe^2+^ under anaerobic conditions) → formation of α-Syn aggregates (especially under oxidizing conditions in the presence of ferritin). This interaction with intracellular Fe^2+^ promotes the aggregation of α-Syn. Aggregated and unaggregated forms of α-Syn can bind to the mitochondrial membranes in dopaminergic cells → decreased mitochondrial membrane potential and increased ROS production. The presence of α-Syn aggregates in the mitochondria impairs their function, leading to increased oxidative damage and cell death [42].	Aggregation of mHTT → inhibition of peroxiredoxin (antioxidant) Protein aggregation → direct formation of free radicals [28,78].	Motor neurons have high metabolic demands and PUFA prone to oxidation in their membranes, which exacerbates the existence of misfolded protein aggregates accumulated in the cytoplasm → oxidative and inflammatory damage. Accumulation of misfolded proteins (mutant SOD1) → increased ROS levels → lipid peroxidation, mitochondrial dysfunction, and oxidative damage. Misfolded proteins not only affect motor neurons, but also have an impact on surrounding glial cells, thereby perpetuating a cycle of oxidative damage and neuroinflammation [37,60,66].

α-Syn: alpha-synuclein; AD: Alzheimer’s disease; AGEs: advanced glycation end products; Al: aluminum; ALS: amyotrophic lateral sclerosis; Apo-E: Apolipoprotein E; APP: amyloid precursor protein; ATP: adenosine triphosphate; Aβ: amyloid beta; BBB: blood–brain barrier; Ca^2+^: calcium ion; C9orf72: chromosome 9 open reading frame 72; Cu: copper; Cu/Zn-SOD: copper/zinc superoxide dismutase; DA: dopamine; DAQs: dopamine quinones; DJ-1: protein deglycase DJ-1 (encoded by the PARK7 gene); DNA: deoxyribonucleic acid; D-loop: displacement loop; EMF: electromagnetic fields; ETC: electron transport chain; Fe: iron; GPx: glutathione peroxidase; GSH: glutathione; GSSG: glutathione disulfide; H_2_O_2_: hydrogen peroxide; HD: Huntington’s disease; Hg: mercury; 4-HNE: 4-hydroxynonenal; HPA: hypothalamic–pituitary–adrenal; IL-1β: interleukin-1 beta; IL: interleukin; LPS: lipopolysaccharides; MDA: malondialdehyde; MetS: metabolic syndrome; Mn: manganese; mHTT: mutant huntingtin; NADPH: nicotinamide adenine dinucleotide phosphate; NF-κB: nuclear factor kappa B; NM: neuromelanin; NLRP3: NOD-like receptor family pyrin domain containing 3; NO: nitric oxide; NOX: NADPH oxidase; O_2_^•–^: superoxide anion; OS: oxidative stress; Pb: lead; PD: Parkinson’s disease; PINK1: PTEN-induced putative kinase 1; PUFA: polyunsaturated fatty acids; RNS: reactive nitrogen species; ROS: reactive oxygen species; SIRT3: sirtuin 3; SOD: superoxide dismutase; SOD1: superoxide dismutase 1; TDP-43: TAR DNA-binding protein 43; TNF-α: tumor necrosis factor alpha; Zn: zinc.

## 3. Olive Oil Composition

### 3.1. Detailed Description of Olive Oil’s Chemical Composition, Focusing on Antioxidants like Oleuropein, Hydroxytyrosol, and Oleocanthal

**Figure 2:**
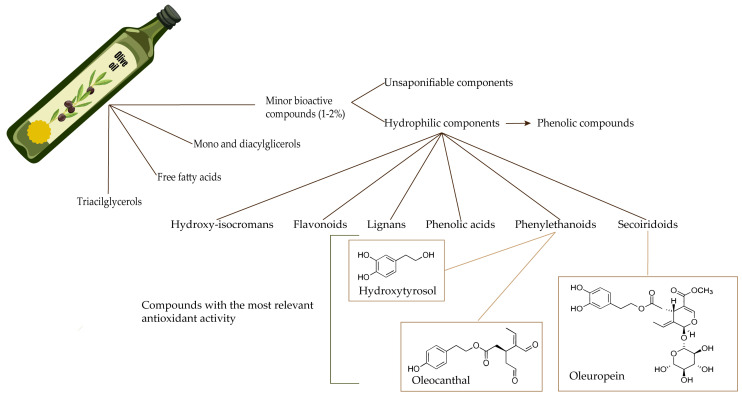
Graphical representation of olive oil composition.

The chemical composition of olive oil can vary depending on multiple factors, such as agronomical and environmental conditions or technological factors involved in processing and storage [79]. However, it is possible to establish a foundational set of components inherent in all varieties of olive oil. As depicted in Figure 2, the main constituents of this ingredient of the MD are triacylglycerols, followed by free fatty acids, mono- and diacylglycerols and, in a lower concentration, minor bioactive compounds, which can be divided into two categories: unsaponifiable and hydrophilic components [80]. The unsaponifiable components are the fraction obtained through saponification of the oil, followed by solvent extraction. On the other hand, the hydrophilic components correspond to the soluble fraction [81]. It is important to mention that olive oil has low concentrations of saturated fatty acids, which contributes to the numerous health benefits associated with this oil [82].

The production of polyols, such as mannitol and oligosaccharides, occurs in the cells of the olive leaf, as a result the of CO_2_ fixation that occurs during photosynthesis. Along with sucrose, these compounds are then transported to the olive, contributing to the overall carbon economy, and allowing for the production of oil and all the molecules that constitute it. Moreover, the olive itself has chloroplasts with the ability of fixing CO_2_ [83]. While photosynthesis can be fundamental in the production of plant’s lipids, glycolysis can play a pivotal role in this process, as the synthesis of fatty acids requires the existence of acetyl-CoA, considering that it forms their carbon backbone. In fact, the pyruvate resultant from glycolysis can serve as a precursor for acetyl-CoA production. Acetyl-CoA can undergo carboxylation to form malonyl-CoA, which constitutes the first step of fatty acids synthesis within the plastids. The malonyl group is subsequently transferred onto the acyl carrier protein and elongation of fatty acids is initiated through the cyclic incorporation of two-carbon units. To form oleic acid, the main fatty acid in olive oil, desaturation needs to occur, considering it is a monounsaturated fatty acid. The addition of carbon units typically ends at C16 or C18 [84]. On the other hand, the synthesis of triacylglycerols is a result of the Kennedy pathway, which involves several reactions that take place in the ER and are catalyzed by acyltransferases [83,84]. Furthermore, incomplete synthesis of triacylglycerols or hydrolytic reactions involving these compounds can lead to the presence of partial acylglycerols, such as mono- or diacylglycerols [85].

As previously mentioned, the minor bioactive compounds encompass two main categories. The unsaponifiable components include hydrocarbons, tocopherol, fatty alcohols, triterpenic alcohols, 4-methylsterols, sterols, other terpenic compounds, and polar pigments, with the most abundant compound being an hydrocarbon that is formed by the condensation of six isoprene units, named squalene [81]. Synthesis of squalene occurs across multiple organelles rather than being restricted to a single one. In fact, it can take place in the cytoplasm, mitochondria, plastids, or ER and via two distinct pathways: the cytosolic mevalonate and the plastidial non-mevalonate pathways, which result in the production of isoprenoid intermediates and, ultimately, squalene [86]. The hydrophilic components include the phenolic compounds, which have several categories, such as secoiridoids, phenylethanoids, phenolic acids, lignans, hydroxyisocromans, and flavonoids [87]. Among these, the secoiridoid oleuropein (OLE) and the phenylethanoids hydroxytyrosol (HT) and oleocanthal (OLC) appear to have the most significant contribution to the antioxidant and anti-inflammatory activities of olive byproducts and, consequently, their health benefits [88,89]. 

#### 3.1.1. Oleuropein

The main phenolic compound in olive fruit is OLE, which is a high molecular weight hydrophilic molecule [90]. In 1908, Bourquelot and Vintilesco firstly demonstrated its existence, describing it as a heterosidic ester of elenolic acid and dihydroxyphenylethanol (or HT), although the chemical structure was only unraveled in 1960 [91,92]. Nevertheless, the concentration of this compound decreases through the course of fruit ripening and processing. Considering this, while most polyphenols in table olives originate from the hydrolysis of OLE, its concentration in edible forms as an isolated glycoside typically remains low [91]. While olive oil contains low levels of OLE, this polyphenol is accountable for its bitterness taste [92,93]. 

**Figure 3:**
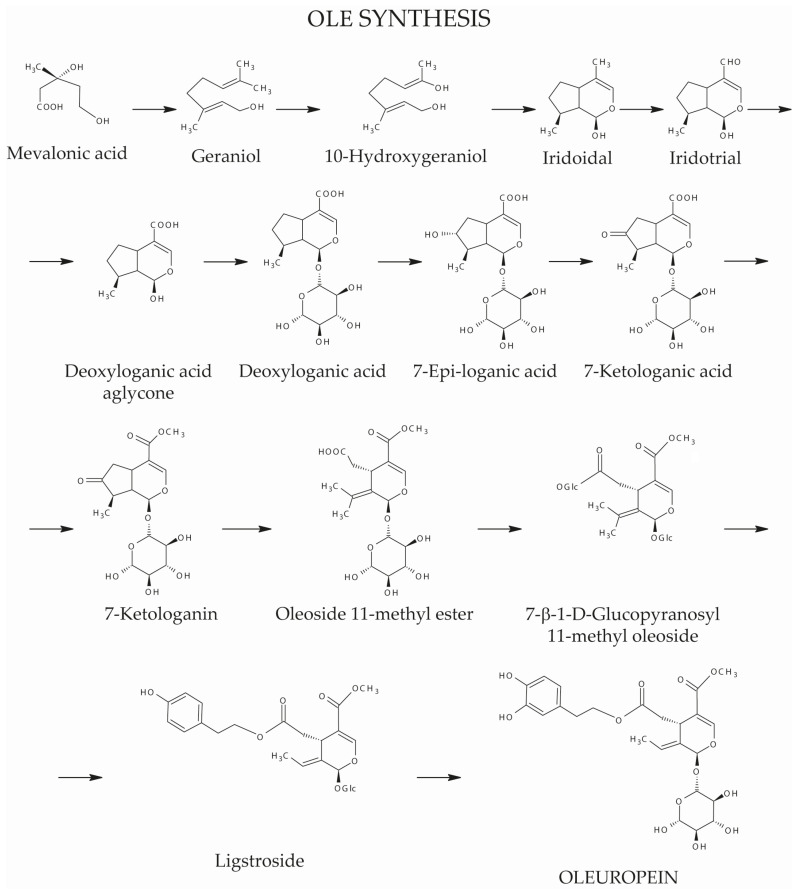
Oleuropein (OLE) synthesis. Adapted from [98].

The molecular structure of OLE is illustrated in Figure 3. It is composed of HT, a monoterpene and a glucose molecule. Moreover, classified as a secoiridoid, OLE is part of a group of compounds that have in common a monoterpene with a heterocyclic ring formed by six carbons and what used to be a cyclopentane ring (as it is in iridoids), but is now opened at the 7,8 bond [92]. Furthermore, mevalonic acid is an important precursor of OLE, since the synthesis of this phenolic compound occurs as a branching event of the mevalonic acid cycle. In fact, mevalonic acid is formed by the condensation of three molecules of acetyl-SCoA, which originates ester β-hydroxy-β-methylglutaryl-CoA and finally, through hydrolysis, mevalonic acid. Figure 3 depicts the biosynthesis of OLE in olives.

In general, polyphenols exhibit decreased bioavailability, as they have low absorption rates and are metabolized and excreted rapidly in the human organism. There are several reasons for this low bioavailability, such as regulatory mechanisms to reduce toxicity, the ability of these compounds to bind to the surface of blood cells and to the microbial flora both in the oral cavity and the gut, as well as their metabolism, which occurs in the gastrointestinal tract and in the liver [94]. The metabolism of phenolic compounds can initiate in the stomach. However, there are some polyphenols that remain in their conjugated form, as they are resistant to gastric hydrolysis due to the conditions of exposure in oil extraction. For instance, when olives undergo crushing and malaxation to originate olive oil, the compounds in their glycoside form are transformed into secoiridoids in an aglyconic form, through hydrolysis of endogenous β-glucosidases. Subsequently, these components continue to be metabolized in the gut, where the microflora can play a fundamental role [95].

Specifically, OLE is a hydrophilic molecule, so it seems unlikely that it crosses the lipid bilayer of cells [90]. In fact, in a study that aimed to evaluate the anti-cancer potential of OLE against human BRAF melanoma cells, it was found that OLE competes with D-glucose for the glucose transporters, indicating this might be the main entrance mechanism of this polyphenol [96]. Moreover, a pharmacokinetic study indicated that OLE was rapidly absorbed and exhibited a biphasic response [97]. Human studies have also demonstrated that the absorption of a group of polyphenols, which included OLE, was as high as 55–66% [95].

In the stomach, acidic hydrolysis can be responsible for the cleavage of the β-glycosidic bond of OLE, thereby releasing glucose and aglycone moiety. On the other hand, the action of the enzyme β-glucosidase can cleave this bond and generate the same metabolites. From these metabolites, two unstable dialdehydes are promptly generated and, at the interface between lipids and water, they are converted into a transposed secoiridoid, a stable lipophilic compound. It is only under severe and prolonged acidic conditions that the two ester groups of the transposed secoiridoid can be cleaved, originating HT and/or methanol. Alternatively, the administration of OLE can occur via gastroresistant capsules, therefore allowing for this compound to be processed in the gut. In the intestine, OLE suffers the action of a lipase that transforms it into HT and methyloleoside. Another lipase can act upon the methyloleoside, resulting in the release of oleoside and methanol [90].

OLE is a potent antioxidant that acts as a free radical scavenger. In fact, it can donate electrons, and the hydroxyl groups that constitute it can donate hydrogens to prevent oxidation. Furthermore, it exhibits metal-chelating activity, preventing the formation of free radicals, which include ROS and RNS [99]. The reduction of intracellular ROS can have an influence on inflammation, since normally ROS control inflammation through activation of NF-KB and AP-1. By blocking these pathways and inhibiting translocation of NF-KB and inflammatory mediators to the nucleus, OLE has a relevant anti-inflammatory effect. This polyphenol also has cardioprotective, hepatoprotective, and neuroprotective activities, as well as anti-cancer and anti-diabetic [99,100].

#### 3.1.2. Hydroxytyrosol

**Figure 4:**
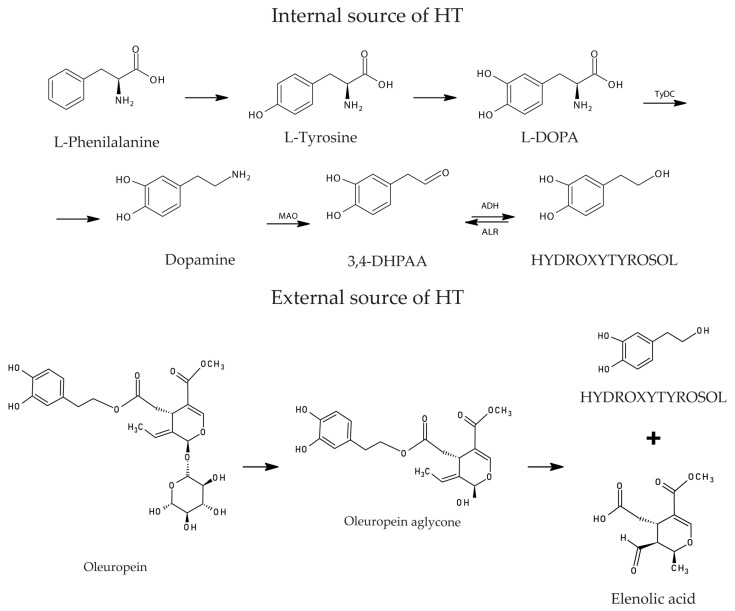
Internal and external metabolism of HT. MAO—monoamino oxidase; ADH—alcohol dehydrogenase; ALR—aldehyde reductase.

HT can result from the hydrolysis of OLE, which can occur during the ripening of the olives as well as throughout their storage and processing into table olives. Hence, it is understandable that it has higher concentration as olives or olives leaves go through maturation and treatment. Contrary to OLE, HT is found abundantly in olive oil in its isolated form, playing a crucial role in guaranteeing its health benefits. It is an amphipathic phenol and can be found it its free form, as an acetate or as part of more complex molecules such as OLE [101,102]. Moreover, as depicted in Figure 4, it has a phenylethyl alcohol structure.

This polyphenol can be originated from an internal source, particularly the dopamine metabolism or an external source, both of which are illustrated in Figure 4 [103,104,105]. The dopamine pathway can initiate with L-phenylalanine, which is converted to L-tyrosine. Subsequently, L-3,4-dihydroxyphenylalanine, the precursor of dopamine, arises from this reaction, allowing for the formation of the neurotransmitter, through the action of a Tyrosine/Dopa decarboxylase. Dopamine is converted into 3,4-DHPAA by MAO. The last step of this metabolic pathway is the generation of HT in a reversible reaction catalyzed by an alcohol dehydrogenase [102,105]. On the other hand, the mechanism that constitutes the external source of HT and occurs in olives as they undergo maturation appears to be simpler. In fact, a β-glycosidase transforms OLE into OLE aglycone, which then, through hydrolysis, originates elenolic acid and HT [102]. 

Polyphenols found in food typically come in two forms: glycosides and aglycones. To be absorbed, the former usually undergo enzymatic deglycosylation to remove the sugar molecule and convert them into absorbable aglycones. On the other hand, glucosides can be transported by the sodium-dependent glucose transporter 1 and further broken down by cytosolic β-glucosidases. In fact, the aglycones are then taken up by enterocytes. However, there are unabsorbed phenolic compounds that reach the colon, where they undergo extensive metabolism by microorganisms [106]. The absorption of HT occurs in the small bowel and colon and depends on the vehicle used, as it is more efficient when the polyphenol is administered within olive oil [102].

After absorption, all polyphenols go through a two-phased metabolism that occurs in the intestine and in the liver. Phase I consists of oxidation, reduction, and hydrolysis (normally occurring under acidic conditions of the stomach) that are catalyzed by the cytochrome P450 family of enzymes, and phase II corresponds to conjugation into glucuronidated, methylated, and sulfated forms [106,107,108]. Nevertheless, free form on HT can still be detected in caecum and feces, indicating it can reach the large intestine. This could potentially be explained by the action of microbial enzymes that deconjugate phase II metabolites [109]. 

Indeed, while free HT is present in human plasma in relatively low concentrations, approximately 98% of HT in glucuronide form is detected in plasma and urine. The reason for this could be the fact that the half-life of HT in plasma is 1–2 min due to rapid metabolism. Moreover, this compounds is able to spread easily to surrounding tissues, as well as crossing the blood–brain barrier [102]. 

HT exerts its antioxidant potential by acting in a similar manner to OLE. Additionally, HT presents particular mechanisms to fight oxidative stress, including the activation of phase II detoxifying enzymes and mitochondrial biogenesis, as well as improving ER biogenesis and activating pathways that allow for adaptation when the ER is under stress [93]. Furthermore, it inhibits the cooper sulfate-induced oxidation of LDL and exhibits anti-inflammatory effects, through the suppression of pro-inflammatory cytokines. Several other health benefits of this polyphenol have been described, which include, among others, anti-cancer, antimicrobial, and anti-diabetic effects [93,110].

#### 3.1.3. Oleocanthal

The secoiridoid OLC was firstly isolated in 1993, where it was described as the dialdehydic form of elenolic acid linked to (ρ-hydroxyphenyl)-ethanol [111]. However, it was only in 2005 that the compound was named “Oleocanthal”, with oleo- for olive, canth- for sting (due to the throat burning sensation that is associated with consumption of this compound and, consequently, olive oil) and -al for aldehyde. The following year, a method for its synthesis was patented [112]. In fact, OLC accounts for only 10% of extra virgin olive oil (EVOO) total polyphenolic content, although its specific concentration can vary with several factors, for instance, olive cultivar. Contrary to other phenolic compounds, OLC presents a high resistance to heating associated with cooking, remaining relatively stable [113]. 

The biosynthesis of this phenolic compound remains poorly documented in the current scientific literature. Nevertheless, there are several synthetic methodologies that have been tested and described [114,115,116]. 

Since OLC is an amphiphilic molecule, it is distributed between the oily and aqueous phase existent in the stomach, considering it is slightly more concentrated in the later, due to the presence of polar functional groups in its chemical structure. In this organ, the phenolic compound is hydrolyzed to tyrosol, although there are studies that indicate that it can maintain stability, therefore not being hydrolyzed. Moreover, the non-hydrolyzed OLC undergoes phase I and phase II metabolism both in the stomach and small intestine. In the liver, CYP enzymes appear to have a significant role in phase I-dependent drug metabolism, therefore being involved in subsequent reactions that occur in metabolites that come from the stomach or intestine. These metabolites are later absorbed in the intestine [117]. In fact, a study that evaluated the biotransformation of this polyphenol found that OLC is metabolized through phase I metabolism, which includes hydroxylation, hydration, and hydrogenation. The metabolites that undergo hydrogenation can further be submitted to phase II metabolism, which might include metabolic reactions, such as glucuronidation [113]. 

In fact, information concerning bioavailability and pharmacokinetics of OLC is still scarce, as most studies reflect upon these properties, but applied to OLE, HT, and tyrosol. Nevertheless, intestinal permeation has been assessed to evaluate the pharmacokinetic of the compound and the results were positive, since both OLC and oleacein obtained a permeation of 50%. It is important to mention that tyrosol achieved a higher value of 78%, indicating its potential higher absorption levels in humans [118]. A distinct study aimed to evaluate the same properties as well, by utilizing LC-HRMS/MS. The findings not only revealed the inability to detect OLC itself in plasma, suggesting a short half-life in vivo, but also unveiled two potential biomarkers of the polyphenol: oleocanthalic acid and tyrosol sulfate. Oleocanthalic acid is a product of phase I metabolism, specifically oxidation, while tyrosol sulfate arises from hydrolysis (phase I metabolism), followed by sulfonation (phase II metabolism) [119].

**Figure 5:**
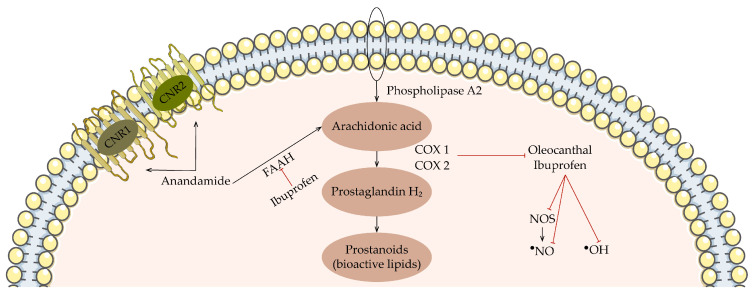
Involvement of ibuprofen and oleocanthal (OLC) in the prostaglandin pathway. Adapted from [123].

OLC is a well-known anti-inflammatory natural compound, whose properties have been compared to those of the non-steroidal anti-inflammatory drug ibuprofen. In fact, this phenolic compound has proved to have even stronger effects against inflammation [120,121]. In fact, both compounds inhibit cyclooxygenase 1 (COX 1) and cyclooxygenase 2 (COX 2), which are part of the prostaglandin pathway, demonstrated in Figure 5 [122]. 

This pathway initiates with arachidonic acid resulting from the action of phospholipase A2 upon phospholipids of the cell membrane. Afterwards, COX 1 and COX 2 convert arachidonic acid into prostaglandin H2, an unstable intermediate [123]. In fact, while COX 1 in generally expressed in tissues, playing an important role in homeostasis, COX2 is expressed in inflammatory cells when stimuli, such as cytokines and bacterial endotoxins, are present. The anti-inflammatory effect of these enzymes is clearly associated with their production of prostaglandins and thromboxane [122]. Prostaglandin is then converted by tissue-specific enzymes into prostanoids, such as PGE_2_, PGD_2_, PGF_2α_, PGI_2_, and TxA_2_, which exert several effects [123]. OLC has similar inhibitory function as that of ibuprofen. Nevertheless, while the phenolic compound is thought to increase p38 phosphorylation, therefore reducing p38 expression, which results in the inhibition of the COX enzymes, ibuprofen binds reversibly to the active site of the same enzymes [122,124]. Additionally, the non-steroidal drug has some other effects that contribute to its overall anti-inflammatory effect. For instance, it can scavenge HO^•^, ^•^NO, and ONOO^−^, which are either ROS or RNS produced by immune cells during inflammation [123]. Interestingly, the first study to evaluate the scavenger capacities of OLC demonstrated its remarkable antioxidant profile, which is actually comparable to that of tyrosol, considering it is capable of scavenging ROS, such as HOCl and O2^•−^ [118]. OLC also has the ability to inhibit the production of inflammatory mediators, which is induced by LPS in macrophages and chondrocytes [113]. Moreover, ibuprofen demonstrated analgesic effects, since it can trigger the antinociceptive axis by interacting with cannabinoid receptors, which include the cannabinoid receptor 1 and cannabinoid receptor 2, and hindering the action of an enzyme named fatty acid amide hydrolase (FAAH), responsible for breaking down the endocannabinoid anandamide. Hence, through the inhibition of FAAH, ibuprofen elevates the levels of anandamide, resulting in enhanced ability to activate cannabinoid receptors and thereby exerting analgesic effects [123]. To our knowledge, no studies have evaluated whether OLC has the same analgesic effects.

Furthermore, OLC has demonstrated remarkable anti-cancer potential, by potentiating apoptosis or having an anti-proliferative effect in colon and breast cancer cells and having anti-melanoma activities. Other health benefits of OLC have been described, such as neuroprotective and anti-rheumatic [113].

### 3.2. Discussion on the Different Types of Olive Oil and Their Relative Antioxidant Capacities

As defined by the EU Council Regulation (EC) No 1234/2007, there are three general types of olive oil, which are virgin olive oils (VOOs), refined olive oil, and olive oil (constituted by a blend of refined and VOOs). Concerning VOOs, they can be defined as oils derived from olives through mechanical or alternative physical methods, therefore not resulting in any chemical and biochemical alterations in the oil. Three main subcategories arise regarding VOOs, considering distinct acidity levels: EVOO, with a maximum free acidity, in terms of oleic acid, of 0.8 g per 100 g; VOO, withholding an acidity level of 2 g per 100 g; and lampante olive oil (LOO), whose acidity level is usually more than 2g per 100 g [125]. On the other hand, refined olive oil, with a free acidity content of not more than 0.3 g per 100 g, is obtained after refining of VOOs, not resulting in changes in the initial glyceridic structure, but rather leading to the removal of most bioactive and antioxidant compounds [126]. The refining process includes neutralization of acidity using alkalis or ion-exchange resins, deodorization through steam injection and/or vacuum application, and bleaching utilizing activated carbon or diatomaceous earth [125,127]. Lastly, the olive oil composed of a mixture of refined olive oil and VOO (except LOO) usually has an acidity level of 1 g per 100 g [125]. In fact, higher-quality oil is usually associated with lower free acidity levels [128].

The oxygen radical absorbance capacity (ORAC) assay is one example of method to assess the antioxidant capacity of compounds since it tests their ability to protect a fluorescent probe from oxidative degeneration [129]. A study utilized this assay to verify the antioxidant capacity of VOO and obtained values in the range of 183–949 µ mol TE/100 g, while a distinct study found that the values for EVOO were between 178 and 620 µ mol TE/100 g [130]. Although we would expect to see higher values for EVOO due to the higher content of phenolic compounds, these results do not illustrate that. Moreover, sensory attributes, such as the bitter and pungent sensation, and antioxidant capacity were found to be positively correlated with EVOO and VOO categories. In fact, the same correlation could not be found in LOO, as the total phenolic content is substantially lower, when comparing with the other two categories [131].

As previously mentioned, obtaining refined olive oil involves the removal of the bioactive compounds; hence, it is expected that this category of olive oil has the lowest antioxidant capacity. The ORAC value for this oil was of 155 µ mol TE/100 g [132].

## 4. Explore How Olive Oil’s Antioxidants Can Potentially Counteract Oxidative Stress in the Brain

Olive oil’s polyphenols exhibit remarkable antioxidant and anti-inflammatory activities which, in theory, makes them appropriate candidates as neuroprotective compounds. Nevertheless, there is an important factor that needs to be considered: whether these phenolic compounds can cross the blood–brain barrier. Various studies have accessed this issue to verify if it could be possible to translate in vitro results into in vivo models and, therefore, validate if these bioactive compounds could exert their neuroprotective activities. For instance, in a study whose aim was to evaluate the ability of HT to cross the endothelium of the blood–brain barrier, the authors found that the percentage of endothelial transport of this polyphenol was of 70% [133]. These findings align with prior research in the field [90,134]. Moreover, regarding OLE, it has been discovered that OLE aglycone is the metabolite of OLE with greater capacity to penetrate the blood–brain barrier, since it can easily cross membranes through passive diffusion [135,136]. Additionally, OLE has been shown to reduce the permeability of the blood–brain barrier, thus contributing significantly to its neuroprotective effects [137]. Finally, mice treated with OLC have an improvement in the cerebral clearance of Aβ through the blood–brain barrier, underscoring its efficacy in traversing the blood-brain barrier [138]. 

Phenolic compounds primarily exert their neuroprotective functions through one central mechanism, the nuclear factor erythroid 2-related factor 2/antioxidant response element (Nrf2/ARE) pathway [139]. In fact, Nrf2 is critical to mitigate oxidative stress in the brain [140]. Hence, in physiological and basal conditions, Nrf2 is inhibited by the Kelch-like ECH-associated protein 1 (Keap1), forming a complex that remains in cytosol until Nrf2 enters the proteasome, where it is ubiquitinated and degraded. In oxidative stress situations, Keap1 senses ARE due to the cysteine residues and Nrf2 is released. Translocation of this transcription factor to the nucleus follows and here it forms heterodimers with the small Maf protein. Nrf2 then binds to ARE that regulates the expression of numerous phase II detoxifying enzymes. Polyphenols can activate Nrf2, therefore contributing to the activation of endogenous antioxidant response [33,139]. Additionally, polyphenols were found to regulate the levels of several neurotrophins, including, for instance, the nerve growth factor. These findings indicate that they can exert neuroprotection by stimulating neuron growth and survival [141].

The neuroprotective efficacy of olive oil’s polyphenols encompasses not only their antioxidant properties, but also their robust anti-inflammatory properties. To understand how preventing inflammation correlates with protection in the tissues of the nervous system, one must firstly comprehend the meaning of hypoxia/reoxygenation and how it can increase the risk of developing neurodegenerative diseases. The brain can experience decreased levels of oxygen compared to those it needs to fully function, which is called hypoxia. This can lead to cell damage. Nevertheless, following this period, reoxygenation can occur and this reintroduction of oxygen can trigger and accentuate cell damage, due to, for example, oxidative stress [142]. Evidently, these events have been associated early on with the development of neurodegenerative pathologies [143]. Hence, VOO was found to inhibit inflammatory mediators that stimulate inducible nitric oxide synthase (iNOS) or interfere directly with the activity of the enzyme, after the brain experiences hypoxia-reoxygenation, reducing overall oxidative stress and brain damage [144]. In fact, either olive oil or olive leaf extracts enriched with certain polyphenols have shown promising results in reducing neuroinflammation [145].

Moreover, these compounds have potential to inhibit apoptosis, including H_2_O_2_ mediated cell death. This capacity is fundamental considering cell death plays an important role in the pathogenesis of neurodegenerative disorders. Polyphenols can induce cytoprotective effects, through the hyperpolarization of the basal mitochondrial membrane potential, as well as decreasing the activity of nerve Na^+^/K^+^ ATPase [146,147].

### 4.1. In Vitro Studies

**Figure 6:**
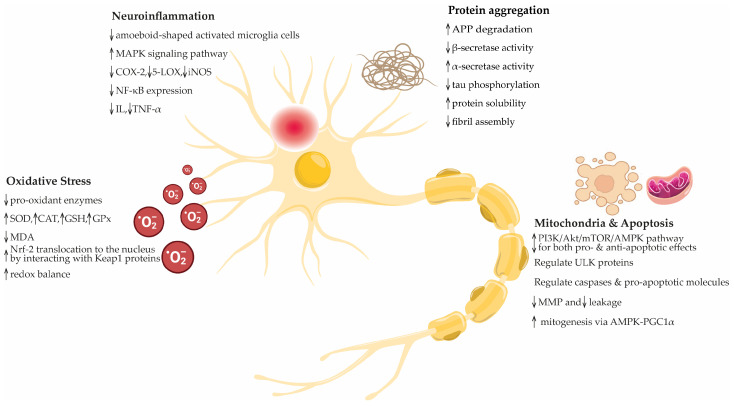
Main mechanisms through which olive oil and its compounds exert their neuroprotective effects, as well as key cell processes involved. APP: amyloid precursor protein; UPS: ubiquitin–proteasome system; SOD: superoxide dismutase; CAT: catalase; GSH: glutathione; GPx: glutathione peroxidase; MDA: malondialdehyde; Nrf-2: nuclear factor erythroid 2-related factor 2; Keap1: Kelch-like ECH-associated protein 1; MAPK: mitogen-activated protein kinase; COX-2: cyclooxygenase-2; 5-LOX: 5-lipoxygenase; iNOS: inducible nitric oxide synthase; NF-κB: nuclear factor kappa-light-chain-enhancer of activated B cells; IL: interleukin; TNF-α: tumor necrosis factor alpha; PI3K: phosphoinositide 3-kinase; Akt: protein kinase B; mTOR: mechanistic target of rapamycin; AMPK: AMP-activated protein kinase; ULK: Unc-51-like autophagy-activating kinase; MMP: mitochondrial membrane potential; PGC1α: peroxisome proliferator-activated receptor gamma coactivator 1-alpha.

Olive oil compounds display significant neuroprotective properties in various in vitro models and employ multiple mechanisms to safeguard neural cells. Specifically, HT and its derivatives have been shown to reduce markers of cell death and oxidative stress. By mitigating lipid peroxidation and preventing the depletion of vital antioxidants, such as glutathione (GSH), these compounds help maintain cellular integrity under stress conditions. Furthermore, olive oil phenolics exhibit considerable anti-inflammatory effects, reducing the expression of inflammatory markers and inhibiting the pathways involved in inflammatory responses. Consequently, the neural cells are protected from inflammation-induced damage. Moreover, these compounds influence cellular signaling pathways, which are critical for cellular defense against oxidative stress. The activation of pathways such as Nrf2/ARE leads to the upregulation of protective enzymes, enhancing the ability of cells to counteract oxidative damage. This dual role of both an antioxidant and a modulator of protective signaling pathways underscores their potential in preventing neurodegeneration. While it is difficult to definitively conclude which pathological context benefits the most from the protective effects of olive oil compounds, such as AD, PD, HD, and ALS, studies indicate broad neuroprotective benefits that could be relevant across these neurodegenerative diseases. Evidence suggests that these compounds can mitigate oxidative stress and inflammation, which are common factors in the pathology of AD, PD, HD, and ALS (Figure 6). Therefore, although the data support the potential of olive oil compounds to provide neuroprotection, further research is necessary to determine whether their effects are more pronounced or beneficial in one specific pathological context. Table 3 summarizes the neuroprotective effects of the olive oil compounds observed in vitro, which are multifaceted and involve antioxidant, anti-inflammatory, and cytoprotective mechanisms. This makes them promising candidates for further research and for potential therapeutic applications in neuroprotection. 

### 4.2. In Vivo Studies

Most in vivo studies have indicated that olive oil compounds, particularly HT, possess neuroprotective properties in various neurodegenerative disease models by reducing oxidative stress, inflammation, and neuronal damage (Table 4). These compounds exhibit potent antioxidant activity by scavenging free radicals, reducing oxidative stress, and inhibiting lipid peroxidation. Additionally, they enhance cellular antioxidant defense mechanisms, including increasing GSH levels and upregulating the activity of antioxidant enzymes, such as GPx and GSH reductase. The protective mechanisms of olive oil compounds involve the modulation of several pathways, including the Nrf2/ARE signaling pathway, which leads to upregulation of cytoprotective enzymes. These compounds also reduce the activity of inflammatory mediators, such as NF-κB and cytokines; inhibit the production of NO; and prevent mitochondrial dysfunction. Studies have demonstrated that these compounds can mitigate oxidative stress, reduce inflammation, and improve overall neuronal health, which are the common pathological features of AD, PD, HD, and ALS. Although further research is necessary to identify any potential disease-specific advantages, the current data suggest that olive oil compounds have a general neuroprotective role that could be beneficial across multiple neurodegenerative conditions. However, the in vivo studies reviewed in Table 4 had several limitations. The diversity of animal models and disease conditions utilized in these studies introduces variability in the results, potentially affecting the generalizability of the findings. It is important to note that different models may not perfectly replicate human disease pathology and the effectiveness observed in animals may not directly translate to humans. Furthermore, the dosages and methods of administration of olive oil compounds vary widely among studies, which complicates the comparability of results and determination of optimal dosing regimens for potential therapeutic use. Many studies have been conducted over relatively short periods; therefore, the long-term effects and potential side effects of chronic administration of these compounds remain poorly understood. Some studies have used combinations of olive oil compounds with other treatments such as levodopa or aspirin. Although these combinations show enhanced effects, it is challenging to isolate the specific contribution of each component. Although these studies have suggested various protective mechanisms, such as antioxidant activity and anti-inflammatory effects, detailed mechanistic insights are often limited. Understanding the precise molecular pathways involved is crucial for the development of targeted therapies. Behavioral improvements have been reported in some studies; however, these assessments can be subjective and vary with different testing methods. More standardized behavioral tests would strengthen the evidence of cognitive and functional benefits. Furthermore, some studies may have limited sample sizes, which could affect the statistical power and robustness of the conclusions drawn. Larger studies with adequate power are required to confirm these findings. Overall, although the results are promising, these limitations highlight the need for further research to validate the therapeutic potential of olive oil compounds in neurodegenerative diseases.

### 4.3. Clinical Trials

One of the first clinical studies examined the association between olive oil consumption, a major component of the Mediterranean diet, and cognitive functioning in people over 65 years over a four-year follow-up. The three-city, multi-center cohort study in Bordeaux, Montpellier, and Dijon focused on estimating dementia and cognitive impairment risks related to vascular factors. Between 1999 and 2001, 9294 individuals were recruited, excluding 217 with prevalent dementia, leaving 9077 participants. By the third round, 7053 participants were examined, with a 4-year follow-up completion rate of 89.1%. The study concludes that in a large non-demented elderly population, intensive olive oil consumption is associated with lower odds of cognitive deficits and decline in specific cognitive functions, independently of other dietary intakes. These findings support the potential cognitive benefits of olive oil [204]. Another similar study, the PREDIMED-NAVARRA randomized trial, suggested that nutritional intervention with a Mediterranean diet supplemented with EVOO or nuts is associated with improved global cognition. This benefit was observed independently of potential confounders such as age, family history of cognitive impairment or dementia, genotype, education, physical activity, vascular risk factors, and energy intake. This is supported by mechanisms related to antioxidative and anti-inflammatory effects, and the reduction of vascular comorbidities by components of this diet, like the EVOO, which have antioxidant properties and are associated with improved cognitive function [205].

Better than a standard Mediterranean diet are the effects of high- (HP-EH-EVOO) or moderate- (MP-EVOO) phenolic EVOO in mild cognitive impairment (MCI). The MICOIL Pilot Study assessed a cohort of Greek elderly patients with MCI; the consumption of Greek HP-EH-EVOO and MP-EVOO was linked to improved cognitive performance over 12 months. Both EVOOs had high phenolic content, which might contribute to their cognitive benefits. The results suggest that Greek EVOO could act as a protective dietary component potentially preventing the progression from MCI to AD. Both HP-EH-EVOO and MP-EVOO are rich in oleic acid, which has been linked to lower inflammatory markers such as C-reactive protein and tumor necrosis factor alpha [206]. Another clinical study (Auburn University Research on Olive Oil for Alzheimer’s Disease (AU-ROOAD)) demonstrated that EVOO reduces blood–brain barrier permeability, reduces levels of the neurotoxin amyloid-B, and improves clinical dementia in a cohort of 25 participants. This barrier is often compromised in MCI patients, which can lead to increases in neurotoxins in the brain, accelerating the disease progression to severe cases of Alzheimer’s and dementia [207]. 

Ongoing clinical studies focusing on the neuroprotective effects of olive oil include using olive leaves that contain phenolic compounds, like oleo-European and HT, to examine and contrast the effects of a Mediterranean diet and olive leaf beverages on memory and cognitive function in people with MCI (GOLDEN-NCT04440020, [208]). Another is the study of Nutraceutical Intervention with High Phenolic Extra Virgin Olive Oil and Curcumin for Neurofibromatosis, Type 1, combining these two compounds on this disease, which causes the growth of tumors in the nerves (NCT05363267, [209]).

The reviewed studies, summarized in Table 5, consistently highlight the significant cognitive benefits associated with olive oil consumption, particularly within the context of the Mediterranean diet. Use of olive oil, especially high-phenolicEVOO, is linked to improved cognitive function and a lower risk of cognitive decline in elderly populations. These findings suggest that olive oil’s antioxidative and anti-inflammatory properties play a crucial role in its neuroprotective effects. The relevance of these findings in healthcare is substantial, given the growing prevalence of neurodegenerative diseases like Alzheimer’s and Parkinson’s.

## 5. Challenges and Future Directions

One of the primary challenges in assessing the neuroprotective effects of polyphenols lies in their limited bioavailability [211]. This results in a clear difficulty to conduct in vivo assays, considering that decreased doses of these compounds are delivered to the tissues. Moreover, translating laboratory findings to clinical application can be a demanding task. Hence, clinical trials to evaluate the effectiveness of olive oil in preventing neurodegenerative diseases and promoting neuroprotection are scarce. One of the reasons for this is that, in laboratory studies, purified compounds are often utilized, which may not accurately reflect the complex matrix and typical dietary consumption of olive oil. While olive oil’s polyphenols have remarkable biological effects, further research should explore several understudied areas, such as specific dosages, long-term effects of supplementation, and the impact of olive oil consumption on different stages of neurodegenerative disorders, as well as attempting to determine more precisely the specific pathological context in which these compounds are beneficial. Therefore, more large-scale and long-term clinical trials are needed to better understand the effects of these compounds and determine optimal amounts for consumption. Olive oil, a readily accessible and natural dietary component, offers a promising preventive strategy to mitigate the risk and progression of neurodegenerative conditions. Integrating olive oil into dietary patterns, particularly through the Mediterranean diet, could serve as an effective public health measure to improve cognitive health and reduce the burden of neurodegenerative disorders. Future research should focus on translating these findings into practical dietary recommendations and clinical applications. This ongoing exploration is crucial for developing comprehensive strategies to combat the rising incidence of neurodegenerative diseases and enhance overall cognitive well-being.

## 6. Conclusions

Neurodegenerative diseases pose a major challenge to global healthcare, as currently, there are no treatment options that aim to prevent neuronal loss. Instead, available therapies only address the symptoms. This underscores the critical need to discover compounds with neuroprotective properties that could potentially prevent or treat these illnesses. Olive oil can be considered the cornerstone of the Mediterranean diet, as its consumption is fundamental in this regimen. In fact, most of olive oil’s health-related benefits are associated with a minor portion of its components, the phenolic compounds. Among these compounds, OLE, HT, and OLC are particularly significant, due to their antioxidant and anti-inflammatory properties, which enable them to exert neuroprotective effects. The onset of neurodegenerative diseases has been closely linked to oxidative stress. Therefore, it is reasonable to assume that compounds with antioxidant potential can help mitigate oxidative stress and potentially reduce its impact, contributing to prevention of disease progression or development. In vivo and in vitro trials reviewed in this paper have confirmed these hypotheses, with the polyphenols showing remarkable results. For instance, in addition to their antioxidant properties, these compounds exhibit cytoprotective and anti-apoptotic effects, and possess the ability to reduce protein aggregation, a process commonly linked to the pathogenesis of neurodegenerative disorders. While there are evident challenges in translating preclinical findings to clinical applications, there is an increasing effort to confirm the notable neuroprotective effects of olive oil in clinical trials. Nevertheless, more large-scale and long-term clinical trials are still needed to better understand the potential of olive oil in mitigating the risk of neurodegenerative diseases. Moreover, continued research into the specific mechanisms underlying olive oil’s role in preventive neurology is fundamental for harnessing its full therapeutic potential and advancing preventive strategies against these conditions.

## Figures and Tables

**Table 1:** Reasons for the susceptibility of the brain to oxidative stress. Adapted from [29].

Reasons for the Brain’s Susceptibility for Oxidative Stress	Examples and Consequences of Radical Formation
Radicals are important in fundamental biological processes.	The O_2_^•−^ and H_2_O_2_ produced by NOX2 are capable of regulating, through the PI3K/Akt signaling pathway, adult hippocampal progenitor cell growth, therefore maintaining neural progenitor cells. NOX isoforms have important functions in processes related with learning and memory. The H_2_O_2_ that is produced by NOX has a role in axonal pathfinding and regeneration. Redox potentials regulate the release of Ca^2+^, fundamental for synaptic plasticity.
The brain relies on Ca^2+^ signaling, which in turn can trigger several neurotoxic cascades that produce reactive species.	Ca^2+^ activates enzymes such as neural NOS, which synthesizes NO^•^ in the presence of O_2_ and NADPH. NO^•^ inhibits COX and, consequently, mitochondrial respiration. NO^•^ + O_2_^•−^ → ONOO^−^; ONOO^−^ + CO_2_ → CO_3_^•−^ + NO_2_^•−^; both CO_3_^•−^ and NO_2_^•−^ can lead to neurodegeneration. Ca^2+^ results in increased phospholipase A2 that, through enzymatic or nonenzymatic pathways, leads to peroxidation of bis-allylic unsaturated lipids. Excessive mitochondrial Ca^2+^ leads to the opening of the permeability transition pore, causing O_2_^•−^ and H_2_O_2_ efflux, and inhibiting ATP synthesis. This can lead to apoptosis and neurodegeneration. Conversely, mitochondrial Ca^2+^ deficiency hinders ATP production and increases NADPH and ROS concentrations.
Glutamate, a fundamental neurotransmitter, can result in decreased endogenous antioxidant defenses and excessive Ca^2+^ influx, which activates harmful neurotoxic pathways that produce radicals.	Excessively high concentrations of glutamate result in overload of Ca^2+^, and therefore O_2_^•−^ and H_2_O_2_ are released from the mitochondria, leading to cell death. Ca^2+^ activates enzymes such as neural NOS, which can inhibit COX and, consequently, increase concentration of O_2_^•−^, H_2_O_2_, and ONOO^−^. Glutamate can inactivate the system Xc—transporter, responsible for transporting cystine into cells, while exporting glutamate. Normally, this exchange allows the synthesis of GSH, meaning the inhibition of the transporter results in OS and ferroptosis, a form of cell death involving Fe and lipid peroxidation.
Neurons have limited capacity to metabolize toxic byproducts of glucose metabolism, which can generate toxicity and cause alterations in proteins, RNA and DNA, leading to OS.	Neurons can convert lactate to pyruvate, although the primary function of glial cells is to metabolize glucose into lactate. While neurons prioritize glucose for the pentose phosphate pathway, they have a limited capacity to metabolize other metabolites, such as methylglyoxal. This lack of enzymes can lead to the toxicity and glycation of proteins, RNA, and DNA, resulting in the formation of advanced glycation end products. These products are associated with inflammation and OS.
Mitochondria are involved in respiration and signaling pathways, both of which produce reactive species.	O_2_^•−^ and H_2_O_2_ are produced in the mitochondria as a result of aerobic respiration. Under hypoxic conditions, mitochondria generate O_2_^•−^ and H_2_O_2_ to activate HIF1-α and initiate a response pathway. At the end of this pathway, HIF1-α can suppress ROS generation under normal circumstances. However, in this situation, aberrant redox signaling can emerge, triggering apoptosis and neurodegeneration.
The metabolism of neurotransmitters might generate H_2_O_2_	Monoamino oxidases are capable of metabolizing neurotransmitters, such as DA, tyramine, tryptamine, and noradrenaline, by catalyzing the following reaction, which generates H_2_O_2_: amine + O_2_ + H_2_O → aldehyde + H_2_O_2_ + NH_3_ For instance, monoamino oxidase B preferentially metabolizes 2-phenylethylamine and is in the inner membrane of the mitochondria, more specifically in the outer face, where the ability to metabolize H_2_O_2_ is reduced. Certain aldehydes generated in neurotransmitter’s metabolism can induce damage as well.
Neurotransmitters, particularly those with catechol groups, can undergo auto-oxidation.	DA+ O_2_ → DA semiquinone radical + O_2_ → DA quinone + O_2_^•−^. The previous reaction can be accelerated by transition metals, abundant in the brain. The DA quinone can engage in redox cycling, therefore generating more ROS.
The brain has relatively low endogenous antioxidant defense mechanisms.	Decreased catalase. Low GSH due to low concentration of Nrf-2, which results in decreased GPx 4 activities; increased ferroptosis and restricted capacity to metabolize electrophiles. PRDX enzymes, which are cysteine-dependent peroxidases, undergo reduction by TRDX, which is then reduced through the action of thioredoxin reductase that utilizes NADPH. These reactions are fundamental in the metabolism of H_2_O_2_. NADPH is used by pro-oxidant enzymes, such as NOS, meaning the molecule is not exclusively used in antioxidant contexts. PRDX6 prefers GSH as a reductant. Hence, low levels of that peptide can limit its activity. It has been suggested that PRDX-TRDX systems allow for neurons to use OS for signaling purposes. Nevertheless, if PDRX becomes over-oxidized due to excessive levels of H_2_O_2_, cell death can occur considering the disrupt in the balance leads to OS.
Transition metals, which are abundant in the brain, can lead to increased levels of reactive species.	Fe^2+^ and Fe^3+^ can regulate the activity of enzymes like aconitase, fumarase, and cytochrome P450. These ions are also cofactors of enzymes responsible for myelin synthesis. Fe^2+^ is associated with ferroptosis, via catalysis of radical generation in a reaction favored when comparing with the Fenton reaction. Neurons have a labile Fe pool that tightly controls the levels of ROS. However, dysregulated Fe homeostasis might lead to OS. Cu^+^ is needed for the activity of several enzymes, such as COX and cooper-zinc SOD, but high concentrations of Cu^+^ can lead to the existence of Fenton reaction, which generates radicals.
The high content of unsaturated lipids in the brain makes it susceptible to lipid peroxidation and OS.	The brain, in contrast to other metabolically active tissues, has a relatively low capacity for β-oxidation of lipids, such as docosahexaenoic acid, an abundant unsaturated fatty acid. This may be due to the organ’s need to conserve O_2_ and avoid generating H_2_O_2_ due to low catalase activity. As a result, docosahexaenoic acid is utilized for signaling pathways. High concentrations of unsaturated fatty acids can cause harm because they contribute to myelin synthesis, which includes fatty acids enriched with cholesterol, which in turn can undergo auto-oxidation through free radical and non-radical pathways. Increased levels of unsaturated fatty acids make cells more vulnerable to lipid peroxidation, which begins when a reactive species generates carbon radicals that react with O_2_ to form ROO. This leads to OS and cell damage. GPx 4 can mitigate lipid peroxidation by transforming ROOH into ROH. the deletion of this enzyme results in ferroptosis. In fact, GPx 4 has decreased activity in the brain, as GSH levels are low.
NOS and NOX are used for signaling.	Neural NOS is the enzyme used to produce NO^•^, which regulates several processes, including axon growth and pruning. NOS can lead to OS not only through the production of NO^•^, but also because, when the enzyme is uncoupled (for instance, due to lack of cofactors), it produces O_2_^•−^ as well. NOX enzymes convert NADPH to O_2_^•−^ and consume O_2_, playing key roles in brain functions such as microglial support and memory/learning. However, OS can also occur.
RNA can undergo oxidation, generating damaging molecules.	RNA oxidation occurs similarly to DNA oxidation, resulting in the formation of 8-oxo-guanine. However, RNA’s single-stranded structure and lack of histones make them more susceptible to oxidation. This oxidation can result in errors in protein synthesis and neurodegeneration.

ATP: adenosine triphosphate; Ca^2+^: calcium ion; CO_3_^•−^: carbonate radical; CO_2_: carbon dioxide; COX: cyclooxygenase; Cu: copper; DA: dopamine; Fe: iron; GSH: glutathione; GPx: glutathione peroxidase; H_2_O: water; H_2_O_2_: hydrogen peroxide; HIF1-α: hypoxia-inducible factor 1-alpha; NADPH: nicotinamide adenine dinucleotide phosphate; NO^•^: nitric oxide radical; NO_2_^•−^: nitrogen dioxide radical; NOS: nitric oxide synthase; NOX: NADPH oxidase; Nrf-2: nuclear factor erythroid 2-related factor 2; O_2_: oxygen; O_2_^•−^: superoxide anion; OS: oxidative stress; ONOO^−^: peroxynitrite; PI3K: phosphoinositide 3-kinase; PRDX: peroxiredoxin; ROH: radical traps; ROO: peroxyl radical; ROOH: lipid hydroperoxide; ROS: reactive oxygen species; SOD: superoxide dismutase; TRDX: thioredoxin.

**Table 3:** Neuroprotection of olive oil compound observed in vitro.

Sample	In Vitro Model	Treatment	Biological Activity	Reference
HT	PC12 cells	PC12 cells were pre-treated with HT at concentrations ranging from 1 to 50 μM for 12 h before being exposed to either H_2_O_2_ or 6-OHDA to induce oxidative damage. Cell viability and other assays were conducted after treatment with H_2_O_2_ or 6-OHDA for various durations, typically 12 to 24 h.	HT scavenged free radicals in vitro, displaying better activity than resveratrol in DPPH assays and protecting erythrocyte ghosts from lipid peroxidation and hemolysis induced by AAPH. HT protected PC12 cells from H_2_O_2_ and 6-OHDA-induced cell damage, maintaining cell viability and reducing LDH leakage. HT decreased the population of apoptotic nuclei and significantly reduced the activation of caspase-3, a key enzyme in the execution phase of apoptosis. HT prevented intracellular ROS accumulation and loss of cellular thiols in PC12 cells treated with H_2_O_2_ or 6-OHDA. HT activated the Nrf2/ARE signaling pathway, leading to the upregulation of cytoprotective enzymes such as HO-1, NQO1, TrxR1, and GCLC and GCLM. The activation of Nrf2 and its translocation to the nucleus were essential for HT’s protective effects, as knockdown of Nrf2 significantly reduced HT’s cytoprotective actions.	[148]
HT	SH-SY-5Y cells	SH-SY-5Y cells were treated with HT at various concentrations (2, 5, and 10 μM) and exposed to high glucose (45 mM) to induce OS. The cells were pre-treated with HT for 24 h before glucose exposure.	HT significantly enhanced cell viability and reduced OS-induced apoptosis in SH-SY-5Y cells. HT increased the expression of HO-1 and complex IV in SH-SY-5Y cells and prevented high glucose-induced ROS production and mitochondrial dysfunction.	[149]
HT	Astrocytic cell line (C6) (AD model)	The astrocytic cell line was exposed to Aβ (25–35) and co-incubated with HT for different periods.	Aβ (25–35) exposure significantly decreased astrocyte viability compared to controls. Both pre-treatment and post-treatment with HT prevented this decrease in viability. HT’s preventive role on Aβ (25–35)-induced cytotoxicity in astrocytes was mediated by an increased HT-induced activation of Akt, part of the insulin signaling pathway. HT prevented the pronounced activation of mTOR, thereby restoring proper insulin signaling. HT protects Aβ (25–35)-treated astrocytes by improving insulin sensitivity and restoring proper insulin signaling.	[150]
HT	BV 2 cells	BV 2 cells were treated with HT at various concentrations (1, 10, 25, and 50 µM) and stimulated with LPS or aggregated α-Syn.	HT demonstrated anti-inflammatory effects by reducing microglial activation. HT decreased the expression of pro-inflammatory markers (TNF-α, iNOS, IL-1β, IL-6, and CXCL10), inhibited the nuclear translocation of NF-κB, and reduced the ROS production via the inhibition of NADPH oxidase. Additionally, HT modulated the MAPK pathways (JNK 1/2, p38, and AKT) involved in the inflammatory response.	[151]
HT and HT-AC (VOO)	Rat brain slices	Brain slices were pre-incubated with HT and HT-AC at various concentrations (1, 5, 10, 50, and 100 µM) during hypoxia and reoxygenation periods.	Both HT and HT-AC significantly reduced LDH efflux, a marker of brain cell death, in a concentration-dependent manner. The IC50 values were 77.78 µM for HT and 28.18 µM for HT-AC.	[152]
HT and its metabolites, which include DOPAL, MOPET, and MOPAL	PC12 cells	The type of administration involves treating PC12 cells with various concentrations of the metabolites (DOPAL, MOPET, and MOPAL) either alone or in combination with α-Syn fibrils. The specific concentrations tested for each metabolite ranged from 0.5 μM to 150 μM.	**DOPAL:** Exhibits the greatest effect in preventing aggregation and α-Syn-induced neurotoxicity, with a potent destabilizing effect on α-Syn fibrils and a significant increase in cell viability. **MOPET:** Effective in inhibiting α-Syn fibril formation and reducing α-Syn-induced toxicity in PC12 cells, though less potent than DOPAL. **MOPAL:** No significant effect on α-Syn fibril formation or destabilization and showed toxicity at higher concentrations. **Gene expression:** Both DOPAL and MOPET significantly increased SIRT-1 and Hsp70 gene expression, and inhibited SIRT-2 gene expression, which are involved in neuroprotective mechanisms.	[153]
HT, Alkyl HT ethers including ethyl, butyl, hexyl, octyl, and dodecyl derivatives	Rat brain slices	Brain slices were incubated with HT and its alkyl ether derivatives at concentrations ranging from 0.5 to 1000 μM. The experimental protocol included: Pre-incubation with the compounds; Induction of hypoxia for 20 min; Reoxygenation with a buffer for a specified period.	**Neuroprotective effects:** All alkyl HT ethers demonstrated neuroprotective effects by reducing LDH efflux. The butyl derivative was the most potent, showing the lowest IC50 values for LDH efflux and lipid peroxidation (TBARS). Antioxidant effects: All compounds inhibited lipid peroxidation in a concentration-dependent manner, with the butyl ether being the most effective. Compounds also mitigated GSH depletion induced by diethylmaleate and prevented OS induced by hypoxia/reoxygenation. HT and its derivatives reduced the levels of PGE2, nitrites plus nitrates (NO_2_ + NO_3_), and IL-1β, indicating anti-inflammatory properties.	[154]
HT, HT-AC, Tyr, DOPAC, DA, and sodium ascorbate	Biomolecular fluorescence complementation technology using *E. coli* BL21 cells	Sodium ascorbate was used to supplement, as it allowed for stabilization by avoiding autoxidation. Specific dosages of HT, HT-AC, Tyr, DOPAC, and DA were administered.	**Reduction in α-syn aggregation:** At low concentrations, most molecules were mildly effective, likely due to oxidation. Increasing the concentration, along with supplementation with sodium ascorbate, improved their effectiveness. In fact, HT-AC and DOPAC were able to inhibit aggregation entirely and DA decreased the aggregation by 28.7%.	[155]
HT, Tyr, and MET	Rat brain tissue	OS was induced with ferrous salts (lipid peroxidation induction), diethyl maleate (depletion of GSH), and hypoxia/reoxygenation in brain slices. The study involved comparing the effects of HT, Tyr, and MET on OS and neuroprotection.	Lipid peroxidation was inhibited in direct proportion to the number of OH groups: HT > Tyr > MET. Exposure to HT led to partial recovery of the GSH system after chemical inhibition or hypoxia/reoxygenation. All three compounds inhibited cell death in hypoxia/reoxygenation experiments, with HT being the most effective. ONOO- formation (3-nitrotyrosine) and inflammatory mediators (PGE2 and IL-1β) were inhibited by all three compounds. The presence of OH groups in these phenolic compounds from VOO is a determinant factor in their antioxidant effect in brain tissue, though this antioxidant effect is not the only explanation for their neuroprotective effect.	[156]
HT, Tyr, CA, and CU extracted from olive mill wastewater derived from the production of four Sicilian EVOOs	Human neuroblastoma LAN5 cells	LAN5 cells were treated with the biophenols (HT, Tyr, CA, and CU) at concentrations of 12.5, 25, 50, and 100 μM for 24 h. Cells were also treated with 40 μM Aβ oligomers alone or in combination with 25 or 50 μM of the biophenols.	**Antioxidant activity:** The antioxidant ability of the biophenols was determined using the ORAC assay. The ORAC values were HT (24,000 μmol TE/g), Tyr (25,000 μmol TE/g), CA (16,000 μmol TE/g), and CU (21,000 μmol TE/g). **Cytotoxicity:** The biophenols were not cytotoxic to LAN5 cells at concentrations up to 50 μM. At 100 μM, a slight decrease in cell viability was observed for HT and CU. **Neuroprotective effects:** The biophenols (HT, Tyr, and CA) demonstrated a neuroprotective role by significantly reducing the oxidative damage induced by Aβ oligomers. CA was the most effective in preserving cell viability and morphology. The biophenols reduced ROS and mitochondrial superoxide production induced by Aβ oligomers. CA showed the most significant reduction in ROS and superoxide levels compared to HT and Tyr.	[157]
HT, Tyr, 3′,4′DHPG, and OLC	Rat brain slices	The brain slices were incubated with HT, Tyr, DHPG, and OLC at various concentrations mimicking those found in EVOO. The polyphenols were administered from the beginning of the experiment (pre-hypoxia) and maintained until the end of the reoxygenation period.	**Cytoprotective effects:** The polyphenol mixture showed significantly greater cytoprotective effects compared to HT alone. The combination reduced LDH efflux. **Antioxidant effects:** The polyphenols demonstrated potent antioxidant effects by reducing lipid peroxidation (TBARS) and inhibiting ONOO- production. **HT and OLC:** Showed the highest antioxidant and cytoprotective potency. **DHPG:** Increased the antioxidant effects of HT at higher concentrations and enhanced its inhibitory effect on ONOO- formation. **Tyr:** Did not significantly modify the antioxidant variables inhibited by HT but showed some synergistic effects in combination.	[158]
Mix 1—OLE, ρ-coumaric, and Tyr; Mix 2—HT, ρ-coumaric, and Tyr	Neuroblastoma cell line (SK-N-SH)	The neuronal cells were exposed to H_2_O_2_ (200 μM) or paraquat and treatment with the mixes at various concentrations followed.	**Radical scavenging capacity:** Mix 1 presented higher capacity of scavenging free radicals, when comparing with Mix 2. Hence, for cellular studies, only Mix 1 was used. **Cytoprotective effects:** Mix 1 at the concentrations of 0.1 and 1 μM allowed for protection of the neuronal cells challenged with H_2_O_2_, while the compounds that constituted it did not have similar effects when used individually. The results were comparable when cells were treated with paraquat, a pesticide that induces OS and is related with PD etiology. **Antioxidant effects:** At 1 μM, Mix 1 reduced the level of ROS by 15% and protected cells from OS related damage, observed through diminished protein carbonyl levels. Moreover, treatment with Mix 1 leads to reduced activation of NF-κB and Nrf2.	[159]
OA	BV 2 cells	BV 2 cells were treated with 7KC at concentrations of 25–50 μM to induce cytotoxic effects, including OS, apoptosis, and autophagy. OA was administered at concentrations of 50, 100, 200, 300, and 600 μM, either alone or in combination with 7KC. Treatments with OA, EA, and DHA were added to the culture medium simultaneously with 7KC for 24 h.	**Cell viability and proliferation:** OA significantly attenuated 7KC-induced inhibition of cell proliferation. **Oxidative stress:** OA reduced 7KC-induced ROS overproduction and lipid peroxidation, as indicated by reduced 4-HNE levels. **Mitochondrial protection:** OA mitigated 7KC-induced loss of mitochondrial transmembrane potential (Δψm) and decreased the percentage of cells with depolarized mitochondria. **Plasma membrane integrity:** OA reduced 7KC-induced plasma membrane permeability, assessed by propidium iodide staining, indicating reduced cell death. **Membrane fluidity:** OA prevented 7KC-induced increase in plasma membrane fluidity, as measured by fluorescence anisotropy using TMA-DPH. **Apoptosis:** OA significantly reduced 7KC-induced apoptosis, evidenced by decreased caspase-3 activation and lower percentage of cells with condensed/fragmented nuclei. **Autophagy:** OA modulated 7KC-induced autophagy, indicated by changes in the LC3-II/LC3-I ratio. OA reduced the autophagic response.	[160]
OLC	Mouse brain endothelial cells (bEnd3 cells) were used as a representative model of BBB	Cells were treated with OLC at concentrations ranging from 0.5 to 50 μM for 72 h.	OLC treatment increased the expression and activity P-gp and LRP1, which are major Aβ transport proteins at the BBB. A significant increase in 125I-Aβ40 degradation due to the upregulation of Aβ-degrading enzymes was observed following OLC treatment.	[161]
OLC	Human astrocytoma cell line (CCF-STTG1). SH-SY5Y cell line, transfected with APP695 (SH-SY5Y-APP) and non-transfected SH-SY5Y cells	Astrocytes were treated with 100 nM of Aβo, 5 μM OLC, or a combination of both for 3 or 7 days. Neuronal cells (SH-SY5Y-APP and SH-SY5Y) were treated with 100 nM Aβo, 5 μM OLC, or a combination for 3 or 7 days. ACM from treated astrocytes was also used to treat neurons.	**Astrocytes:** OLC reduced the baseline and Aβo-induced levels of IL-6 and GFAP in astrocytes. OLC restored the Aβo-induced downregulation of GLT1 and GLUT1 in astrocytes. Astrocytes efficiently took up Aβ monomers and oligomers, with OLC not significantly altering the degradation of Aβ monomers. Neuronal cells: OLC prevented the Aβo-induced downregulation of synaptic proteins PSD-95 and SNAP-25 in SH-SY5Y-APP cells and increased their baseline expression. OLC directly induced the expression of synaptic proteins in neurons without mediation by astrocytes, as ACM from OLC-treated astrocytes did not alter synaptic protein levels in neurons.	[162]
OLE	Human glioblastoma cells (U87)	U87 cells were pre-treated with OLE essential oil at a concentration of 10 µM. After 30 min of OLE pre-treatment, 100 µM H_2_O_2_ was added to induce OS, and the cells were incubated for 3 h.	**Cell viability:** OLE pre-treatment significantly prevented cell losses caused by H_2_O_2_. **GSH levels:** OLE regenerated total antioxidant capacity and GSH levels, which were decreased by H_2_O_2_ exposure. **Nitric oxide and total oxidant capacity:** OLE administration decreased NO and total oxidant capacity levels in treated cells. **iNOS expression:** The relative gene expression level of inducible iNOS was reduced by OLE pre-treatment.	[163]
OLE and olive leaf extract	PC12 cells	PC12 cells were treated with 6-OHDA at a concentration of 150 μM to induce cell damage. Olive leaf extract was administered at doses of 400 and 600 µg/mL. OLE was administered at doses of 20 and 25 µg/mL. Olive leaf extract and OLE were added to the cells 20 min before 6-OHDA treatment and incubated for 24 h.	Olive leaf extract and OLE significantly increased cell viability and reduced 6-OHDA-induced cytotoxicity in PC12 cells. Treatment with olive leaf extract and OLE reduced intracellular ROS levels in 6-OHDA-treated cells. OLE decreased the activation of caspase-3 and balanced the Bax/Bcl-2 ratio, indicating inhibition of apoptosis. Olive leaf extract and OLE also prevented DNA fragmentation induced by 6-OHDA, further confirming their protective effects against apoptosis.	[164]
OLE, Tyr, and CU	SK-N-SH cells	SK-N-SH cells were treated with H₂O₂ to induce OS. Cells were then treated with very low concentrations (1 and 5 nM) of oxidized OLE and oxidized mixtures (Mix) of the three polyphenols for 24 h to evaluate their neuroprotective properties.	**Antioxidant activity:** Significant neuroprotection by oxidized OLE and the oxidized mix against H₂O₂-induced toxicity was observed in SK-N-SH cells. **Neuroprotective effects:** Oxidized OLE and the oxidized polyphenol mix significantly reduced intracellular ROS levels and protein carbonyl levels in neuronal cells exposed to OS. The combination of OLE with Tyr and CU acid showed enhanced neuroprotective effects compared to the individual polyphenols, suggesting synergistic interactions among the compounds. **Mechanism of action:** The neuroprotective effects of the oxidized polyphenols were attributed to their ability to modulate redox signaling pathways and reduce oxidative damage. The presence of oxidized products, such as quinones and dimers, formed during the oxidation process, contributed to the enhanced antioxidant and neuroprotective properties of the mixtures.	[165]
OleA	SH-SY5Y cells	SH-SY5Y cells were treated with α-Syn aggregates in the presence or absence of OleA at a ratio of 1:10 (α-Syn/OleA) for various incubation times (24 h and 5 days).	**Anti-amyloidogenic effects:** OleA interfered with α-Syn aggregation, stabilizing monomeric α-Syn and hampering the growth of toxic oligomers. OleA favored the formation of stable and harmless α-Syn aggregates, reducing the formation of cytotoxic amyloid fibrils. **Reduction in cytotoxicity:** OleA-treated α-Syn aggregates showed reduced cytotoxicity in SH-SY5Y cells compared to untreated aggregates. Higher cell viability was observed when cells were treated with α-Syn aggregates formed in the presence of OleA. **Oxidative stress reduction:** OleA decreased the ability of α-Syn aggregates to induce ROS production in SH-SY5Y cells. ROS levels were significantly lower in cells treated with OleA-formed aggregates compared to those treated with untreated α-Syn aggregates. **Interaction with cell membrane:** OleA reduced the binding of α-Syn aggregates to cell membrane components, particularly to lipid raft-associated ganglioside GM1. This reduction in membrane interaction likely contributes to the decreased cytotoxicity in OleA-treated aggregates.	[166]
OleA	SH-SY5Y cells	OleA was dissolved in DMSO and used in various assays to investigate its effect on protein aggregation and cytotoxicity.	OleA was found to interfere with the aggregation of Syn by stabilizing Syn monomers and preventing the formation of cytotoxic oligomers. It reduced the cytotoxicity of Syn aggregates by preventing their binding to cell membranes and reducing oxidative damage to cells.	[167]
OleA	SH-SY5Y cells	OleA was used to investigate its effect on pE3-Aβ aggregation and cytotoxicity in SH-SY5Y cells.	OleA was shown to reduce the burden of pE3-Aβ by interfering with its aggregation path, reducing its cytotoxicity, and promoting the formation of non-toxic aggregates.	[167]
Phenol fraction extracted from commercial EVOO	HEK cells expressing TLR4 (HEK-Blue-4™ cells). Spinal cord primary cultures from SOD1 mutated (SOD1 G93A) mice	The phenolic extract from EVOO was added to the cell cultures to inhibit TLR4 activation. The dose-dependent inhibition of TLR4 activation was determined with concentrations resulting in an IC50 of about 20 μg/mL.	EVOO phenols inhibited the activation of TLR4 in HEK cells and reduced the release of NO from activated glia. They protected motoneurons from LPS-induced lethality in spinal cord cultures and counteracted motoneuron death induced by SOD1 mutant glia. EVOO phenols demonstrated recognized antioxidant properties, which contributed to their anti-inflammatory and neuroprotective effects.	[168]
Ty and OH-Tyr	N2a cells	Tyr and OH-Tyr were applied to cultured N2a cells in vitro. Aβ was used to induce toxicity in the cells (100 μg/mL).	**Neuroprotection:** Tyr and OH-Tyr decreased cell death when co-treated with Aβ. **Mechanism of Action:** Both compounds attenuated the increase in nuclear translocation of the NF-κB subunits after Aβ exposure. **GSH Levels:** Neither Tyr and OH-Tyr prevented the decrease in GSH induced by H_2_O_2_ or Aβ. **Transcription Factor Activation:** The activation of NF-κB by Aβ was reduced in the presence of Tyr and OH-Tyr.	[169]
Tyr, HT, (−)-OLC, (−)-hydroxyoleocanthal, ligstroside aglycone, OleA, OLE, and (+)-1-acetoxypinoresinol (AC), PN	Schwann cells	The administration involved treating Schwann cells with penitrem A at its IC50 value (20 μM) and then adding the tested olive phenolics.	**Tyr:** No protective activity against Penitrem A at any dose. **HT:** Moderate protective activity, showing 32% toxicity recovery at 10 μM. **Secoiridoids:** (−)-OLC, (−)-hydroxyoleocanthal, and ligstroside aglycone did not show any protective activity. OleA and OLE had modest recovery (10%) at 10 μM. **Lignans:** Consistent and significant protection ratios at different doses. AC and PN significantly increased Schwann cells’ survival rates with notable dose-dependent protection against penitrem A toxicity.	[170]

α-Syn: alpha-synuclein; AAPH: 2,2′-azobis(2-amidinopropane) dihydrochloride; AD: Alzheimer’s disease; Aβ: amyloid beta; Aβo: amyloid-β oligomer; AC: (+)-1-acetoxypinoresinol; pE3-Aβ: pyroglutamylated-3 Aβ; ACM: astrocyte-conditioned media; Akt: protein kinase B; ALS: amyotrophic lateral sclerosis; APP: amyloid precursor protein; ARE: antioxidant response element; BBB: blood–brain barrier; BV 2: murine microglial cell line, CA: caffeic acid; C9orf72: chromosome 9 open reading frame 72; CXCL10: interferon γ-induced protein 10; CU: p-coumaric; DA: dopamine; DHA: docosahexaenoic acid; DHPG: 3,4-dihydroxyphenylglycol; DMSO: dimethyl sulfoxide; DOPAC: 3,4-dihydroxyphenylacetic acid; DOPAL: 3,4-dihydroxyphenylacetaldehyde; DPPH: 2,2-Diphenyl-1-Picrylhydrazyl; EA: elaidic acid; EVOO: extra virgin olive oil; GCLC: glutamate–cysteine ligase catalytic subunit; GCLM: glutamate–cysteine ligase modifier subunit; GFAP: glial fibrillary acidic protein; GLT1: glutamate transporter 1; GLUT1: glucose transporter 1; GSH: glutathione; H_2_O_2_: hydrogen peroxide; HO-1: heme oxygenase-1; HD: Huntington’s disease; HNE: 4-hydroxynonenal; Hsp70: heat shock protein 70; HT: hydroxytyrosol; HT-AC: hydroxytyrosol acetate; IC50: half-maximal inhibitory concentration; IL: interleukin; iNOS: inducible nitric oxide synthase; JNK: Jun N-terminal kinase; 7KC: 7-ketocholesterol; LC3: microtubule-associated protein 1A/1B-light chain 3; LDH: lactate dehydrogenase; LPS: lipopolysaccharides; LRP1: low density lipoprotein receptor-related protein 1; MAPK: mitogen-activated protein kinase; MET: 3,4-di-ortho-methylidene-hydroxytyrosol ethyl ether; MOPAL: 3-methoxy-4-hydroxyphenylacetaldehyde; MOPET: 4-hydroxy-3-methoxyphenethanol; mHTT: mutant huntingtin; mTOR: mammalian target of rapamycin; N2a: fast-growing mouse neuroblastoma cell line; NADPH: nicotinamide adenine dinucleotide phosphate; NF-κB: nuclear factor kappa B; NMDA: N-methyl-D-aspartate; NQO1: NAD(P)H quinone dehydrogenase 1; Nrf2: nuclear factor erythroid 2-related factor 2; OA: oleic acid; 6-OHDA: 6-hydroxydopamine; OLC: oleocanthal; OleA: oleuropein aglycone; OLE: oleuropein; ONOO-: peroxynitrite; ORAC: oxygen radical absorbance capacity; OS: oxidative stress; PD: Parkinson’s disease; PA: penitrem A; PC12: pheochromocytoma cell line; P-gp: P-glycoprotein; PGE2: prostaglandin E2; PN: (+)-pinoresinol; PSD-95: postsynaptic density protein 95; ROS: reactive oxygen species; SH-SY5Y: human neuroblastoma cell line; SIRT: sirtuin; SNAP-25: synaptosomal-associated protein of 25 kDa; SOD: superoxide dismutase; TBARS: thiobarbituric acid-reactive substances; TE: Trolox equivalent; TLR4: Toll-like receptor 4; TMA-DPH: trimethylammonium diphenylhexatriene; TNF-α: tumor necrosis factor alpha; TrxR1: thioredoxin reductase 1; Tyr: tyrosol; VOO: virgin olive oil.

**Table 4:** Neuroprotection of olive oil compound observed in vivo.

Sample	In Vivo Model	Treatment	Biological Activity	Reference
EVOO	Transgenic SOD1G93A mice (ALS model).	Mice were fed a chow diet enriched with 20% (*w*/*w*) EVOO.	Mice fed an EVOO diet showed significantly higher survival and better motor performance compared to control mice. EVOO group mice also had larger muscle fiber areas than those receiving palm oil. The study found a decrease in markers of ER stress, such as Atf6 and Grp78, in mice receiving the EVOO diet. The beneficial effects of EVOO were associated with improved ER stress response and autophagy in muscle tissues.	[171]
EVOO	TgSwDI mice: A transgenic mouse model used to study AD and cerebral amyloid angiopathy.	Mice were fed an EVOO-enriched diet for either 3 or 6 months.	Reduction in Aβ and Tau pathologies: Long-term consumption of an EVOO-enriched diet (6 months) significantly reduced total Aβ and tau levels in the brain. **Cognitive improvement:** There was a significant improvement in mouse cognitive behavior with long-term EVOO consumption. **Enhanced clearance and reduced production of Aβ:** The reduction in brain Aβ was attributed to enhanced clearance pathways and reduced production via modulation of APP processing. Time-dependent effects: Short-term EVOO consumption (3 months) improved Aβ clearance and reduced Aβ levels but did not affect tau levels or cognitive functions.	[172]
EVOO	Acute parkinsonism murine model in C57BL6 mice induced by MPTP.	EVOO, corn oil, and levodopa were administered to the mice.	MPTP allowed for motor disruption in the mice. **Enhanced locomotor activity:** Mice treated with EVOO showed an increase in motor activity, compared to that registered when levodopa was used. **Increased neural survival in the substantia nigra and striatum:** Through immunohistochemistry, it was possible to observe that the group treated with EVOO had lower levels of neuronal loss, compared to the levels registered in the group treated with levodopa. Corn oil did not exhibit similar motor and neuroprotective effects.	[173]
EVOO OOLF OOHF	Rat	2,4-D pesticide, EVOO, and its fractions were administered to rats by gavage for four consecutive weeks.	**OS reduction:** EVOO and its fractions partly reversed the OS caused by 2,4-D pesticide, as indicated by changes in brain lipid peroxide levels, AChE activity, and antioxidant enzyme activities. Restoration of fatty acid composition: EVOO enhanced neuroprotection by restoring brain fatty acid composition, especially the level of DHA.	[174]
EVOO	3xTg mice (AD model)	Mice were fed a regular chow diet supplemented with EVOO starting at 6 months of age for 6 months.	**Amelioration of behavioral deficits:** Mice receiving the EVOO-rich diet showed improvements in their behavioral deficits. **Increase in synaptophysin levels:** There was a significant increase in the steady-state levels of synaptophysin, a protein marker of synaptic integrity. **Reduction in Aβ and tau pathologies:** Significant reduction in insoluble Aβ peptide levels and deposition. Lower amounts of phosphorylated tau protein at specific epitopes. **Activation of cell autophagy:** The reduction in Aβ and tau pathology was secondary to the activation of cell autophagy.	[175]
EVOO	The in vivo model used in this study is the hTau mouse model, which is a mouse model of tauopathy.	The treatment involved dietary administration of eEVOO.	The study found that EVOO directly improves synaptic activity, short-term plasticity, and memory while decreasing tau neuropathology in the hTau mice. Specifically, EVOO consumption ameliorated spatial learning and recognition memory ability, as well as enhanced hippocampal synaptic function and plasticity.	[176]
EVOO	Sprague–Dawley adult male rats Condition: AD model induced by aluminum chloride (AlCl₃)	AlCl₃ at 100 mg/kg administered orally for 15 days to induce AD pathology. Treatments administered for 21 days following the induction of AD: EVOO Rosmarinic acid Donepezil (used as a reference treatment)	**Cognitive impairment:** Cognitive impairment was observed after 15 days of AlCl₃ administration. **Preventive effects:** Treatment protocols with EVOO, rosmarinic acid, and donepezil prevented the occurrence of AD pathology histopathologically. **Oxidative damage:** The study demonstrated that oxidative damage was mitigated by the treatments. **Neuroprotection:** EVOO and rosmarinic acid showed neuroprotective effects similar to donepezil.	[177]
EVOO	Wistar rat strain	EVOO was administered in two dosages: 0.5 mL/kg body weight 2 mL/kg body weight The treatment was conducted over a period of 14 days.	**Neuroprotection:** EVOO showed potential neuroprotective effects by inhibiting ROS. **Modulation of Hsp27:** EVOO significantly downregulated the expression of Hsp27 in the cerebral cortex. **Cellular composition:** There were significant differences in neuron and glia nuclei numbers between groups treated with EVOO and control groups. **OS reduction:** The bioactive components in EVOO, including tocopherols and phenolic compounds, contribute to its antioxidant properties, reducing OS in brain tissue.	[178]
EVOO and HT	Rat model where HD-like conditions were induced using 3NP.	EVOO was administered orally to the rats. The study compares different timing of EVOO administration (before or after 3NP exposure) to assess the optimal protective effects against OS.	EVOO demonstrated significant antioxidative effects, evidenced by an increase in cellular GSH levels and a decrease in oxidative damage. The administration of EVOO both before and after 3NP exposure showed a protective effect against the OS induced in the brain, particularly in the striatum.	[179]
EVOO and OleA	TgCRND8 mice (AD model)	Continuous intake: High doses of OleA were administered, potentially as a nutraceutical or food integrator.	The study supports the beneficial effects of EVOO and highlights that continuous intake of high doses of OleA may prevent or delay the onset of AD and reduce the severity of its symptoms. Mechanisms of action includes autophagy and adult hippocampal neurogenesis, potentially leading to neuroprotective effects.	[180]
EVOO OLC	5xFAD (AD model)	The administration method involved feeding the homozygous 5xFAD mice with refined olive oil, OLC-low EVOO (0.5 mg total phenolic content/kg), and OLC (0.5 mg OLC/kg) for three months.	Both EVOO and OLC significantly reduced the levels of soluble APP and increased the expression of synaptic markers (PSD-95, SNAP-25, and synapsin-1) in the 5xFAD mouse brains. EVOO and OLC demonstrated significant reductions in astrocyte activation, suggesting a reduction in neuroinflammation. The findings suggest that both EVOO and OLC have neuroprotective effects in reducing Aβ production and neuroinflammation while enhancing synaptic health in the context of AD.	[181]
HT	Rat model, specifically focusing on the offspring of rats that were exposed to prenatal stress.	HT was administered at doses of 10 and 50 mg/kg/day.	**Neurogenesis and cognitive function:** HT supplementation improved learning capacity and memory performance in prenatally stressed offspring. HT prevented the stress-induced downregulation of neural proteins, including BDNF, GAP43, synaptophysin, NMDAR1, NMDANR2A, and NMDANR2B. HT increased the expression of the glucocorticoid receptor in prenatally stressed rats. OS and mitochondrial dysfunction: HT alleviated OS and mitochondrial dysfunction in prenatally stressed rats. HT significantly increased transcription factors FOXO1 and FOXO3, as well as phase II enzyme-related proteins, including Nrf2 and HO-1.	[182]
HT	Type-1-like diabetic rats	Specific dosages of DHPG and HT were administered.	**Neuroprotective effects:** HT demonstrated significant neuroprotective effects in the rat model of type-1 diabetes. **Antioxidant activity:** HT exerted strong antioxidant effects, helping to reduce OS in brain tissue. **Anti-nitrosative stress:** HT also contributed to reducing nitrosative stress, which is crucial for protecting neuronal cells. **Synergistic effects:** The combination of HT and DHPG showed enhanced neuroprotective effects compared to each compound alone. This synergy likely results from their combined antioxidant and anti-nitrosative properties. **Protection against retinal damage:** The study found that the combination of HT and DHPG helped mitigate retinal nerve cell damage, which is important for preventing diabetic retinopathy.	[183]
HT	*Caenorhabditis elegans*	The treatment involved administering 100 µg/mL of the HT-rich olive extract to *Caenorhabditis elegans*.	The study demonstrated that the HT-rich olive extract prevents oxidative stress and delays Aβ-induced paralysis in *Caenorhabditis elegans*. The extract reduced the presence of Aβ aggregates and showed the ability to mitigate proteotoxicity associated with tau protein aggregation. Additionally, the extract exhibited antioxidant properties and enhanced stress resistance in the nematodes, contributing to prolonged longevity and improved health span. Gene reporter strains of *Caenorhabditis elegans* indicated that the extract influenced various stress-related pathways, such as those involving the DAF-16/FOXO, SKN-1/NRF2, and Hsp-16.2 proteins.	[184]
HT	C57BL/6 mice (AD model)	AD-like neurotoxicity was induced in mice by intracerebroventricular injection of soluble oligomeric amyloid β1-42 plus ibotenic acid (oA42i). Treatment: HT was administered orally at a dose of 10 mg/kg/day for 14 days. The administration was performed via oral gavage, and the treatment was initiated 4 days post-surgery for inducing AD-like pathology.	**Neuroprotective and cognitive enhancing effects:** Spatial memory improvement: HT treatment significantly improved spatial reference and working memory in oA42i-intoxicated mice, as assessed by radial arm maze performance. Molecular mechanisms: MAPK signaling pathways: HT reversed oA42i-induced downregulation of ERK1/2 and RSK2 phosphorylation and inhibited the activation of JNK and p38, which are associated with neuronal death. PI3K/Akt and JAK2/STAT3 pathways: HT restored the phosphorylation levels of Akt1, JAK2, and STAT3, promoting neuronal survival. Apoptosis inhibition: HT treatment modulated the Bcl-2/Bad ratio, reduced cytochrome c release, and decreased the activation of caspase-9 and caspase-3, thereby inhibiting mitochondria-mediated apoptosis. Gene expression: HT upregulated the expression of SIRT1, CREB, and CREB-target genes (*BDNF*, *c-Fos*, *Nurr1*, and *Egr1*), which are involved in neuronal survival and memory functions. Mitochondrial protection: HT preserved mitochondrial integrity, preventing the structural damage observed in the oA42i group.	[185]
HT	Male db/db mice on a C57BL/6J	Mice were divided into three groups: control, low-dose HT (10 mg/kg/day), and high-dose HT (50 mg/kg/day). HT was administered orally for 8 weeks.	**Mitochondrial function:** HT improved the expression and activity of mitochondrial respiratory chain complexes I, II, and IV in the brain of db/db mice. Specifically, complex I activity was significantly increased. HT treatment enhanced mitochondrial biogenesis and function, indicated by increased expression of PGC-1α and activation of AMPK and SIRT1 pathways. Antioxidant activity: HT induced phase II antioxidant enzymes, including HO-1, SOD1, and SOD2, in the brain of db/db mice. This was associated with a significant reduction in protein oxidation levels. HT improved neuronal survival and function in db/db mice, as evidenced by increased mRNA levels of neuronal markers such as activity-regulated cytoskeleton-associated protein, NMDAR1, and NGF.	[149]
HTEE, tTEE MHTEE	Rat	Rats were treated orally with doses of 10 or 20 mg/kg/day of HTEE, TEE, or MHTEE.	Lipid peroxidation was inhibited by HTEE, TEE, and MHTEE. All three compounds showed similar neuroprotective effects, reducing arbitrary units from baseline measurements. Inhibition of 3-nitrotyrosine production was observed for all three compounds. PGE2 production was reduced in the treated groups. Only HTEE inhibited IL-1β production.	[186]
HT, HT-AC, Tyr, DOPAC, DA, and sodium ascorbate	*Caenorhabditis elegans* NL5901 mutant strain	Sodium ascorbate was used to supplement, as it allowed for stabilization by avoiding autoxidation. Specific dosages of HT, HT-AC, Tyr, DOPAC, and DA were administered.	**Reduction in α-Syn aggregation:** DOPAC and HT-AC were able to decrease the aggregation by 79.2% and 76.2%, respectively. These compounds exhibited a positive effect on the longevity of *Caenorhabditis elegans*, as well. DA was also able to reduce the aggregation.	[155]
HT, nitrohydroxytyrosol, nitrohydroxytyrosol acetate, and ethyl nitrohydroxytyrosol ether	Rat model	The animals received either an acute (single dose; 20 mg/kg, intraperitoneally) or a chronic (one daily dose for 5 days; 20 mg/kg) treatment of HT and its nitro derivatives.	The treatments produced a significant increase in the intracellular levels of DA and its metabolite, DOPAC. The increase in DA levels was similar to that seen with the commercial COMT inhibitor, Ro41-0960. Chronic treatment effects were stronger than acute treatment effects. Unlike Ro41-0960, HT and its derivatives did not produce a significant decrease in the intracellular level of homovanillic acid, although the chronic treatment effect was stronger than the acute treatment effect. These results suggest that these compounds could inhibit catechol-O-methyltransferase activity.	[187]
HT-AC	APP/PS1 (AD model).	The administration method involved oral administration of HT-AC to the transgenic mice.	The study found that HT-AC improved cognitive functions in AD mice, specifically enhancing escape latency, escape distance, and the number of platform crossings in the water maze test. The beneficial effects included amelioration of neuronal apoptosis and reduction in inflammatory cytokine levels. HT-AC stimulated the transcription of ERβ, enhancing neuronal viability and electrophysiological activity in primary neurons. These effects were abolished when ERβ was deficient, indicating the involvement of ERβ in mediating the neuroprotective effects of HT-AC.	[188]
OLC	TgSwDI mice (AD model).	The mice were treated with OLC for 4 weeks.	**Amyloid load:** OLC treatment significantly decreased amyloid load in the hippocampal parenchyma and microvessels. **Cerebral clearance:** This reduction was associated with enhanced cerebral clearance of Aβ across the BBB. **Proteins involved in clearance:** OLC increased the expression of important amyloid clearance proteins at the BBB, including P-gp and LRP1. It also activated the ApoE-dependent amyloid clearance pathway in the mice’s brains. **Anti-inflammatory effects:** OLC reduced astrocyte activation and IL-1β levels in the brains of these mice. **Mechanistic studies:** Demonstrated OLC’s ability to increase P-gp and LRP1 expression, and to enhance the ApoE-dependent clearance pathway.	[138]
OLC	5xFAD mouse (AD model)	Dietary intake: EVOO was consumed as a medical food. Combination treatment: EVOO was combined with donepezil, an acetylcholine esterase inhibitor approved for all stages of AD.	EVOO consumption combined with donepezil significantly reduced Aβ load and related pathological changes. Enhancement in Aβ clearance pathways, including BBB clearance and enzymatic degradation. Shifting APP processing toward the non-amyloidogenic pathway. Improved synaptic function. Strengthened BBB integrity. Decreased inflammation associated with Aβ pathology.	[189]
OLC	Wistar albino rat model of traumatic brain injury.	The study used 26 adults male Wistar albino rats. The rats were divided into four groups: Group 1: Sham group (n = 5). Group 2: Trauma group treated with 10 mg/kg saline intraperitoneally (IP) twice a day (n = 5). Group 3: Rats treated with 10 mg/kg OLC IP twice a day (n = 8). Group 4: Rats treated with 30 mg/kg OLC IP twice a day (n = 8). Brain samples were collected 72 h after injury.	OLC treatment showed neuroprotective effects by targeting secondary injury mechanisms, reducing OS, and inflammation, and promoting tissue repair and recovery in a dose-dependent manner.	[190]
OLC	Wild-type C57BL/6 mice	Wild-type C57BL/6 mice were administered OLC intraperitoneally at a dose of 10 mg/kg twice daily for 2 weeks.	Enhanced clearance of 125I-Aβ40 from the brain was observed in OLC-treated mice, with an increase in brain efflux index(%) from 62.0 ± 3.0% (control) to 79.9 ± 1.6% (OLC-treated). OLC has anti-inflammatory properties similar to ibuprofen, inhibiting inflammatory markers and reducing OS.	[161]
OLC-rich EVOO.	TgSwDI (AD model)	OLC-rich EVOO was administered starting at the age of 6 months for a treatment duration of 3 months.	**Neuroinflammation:** OC-rich EVOO reduced neuroinflammation by inhibiting the NLRP3 inflammasome. **Autophagy:** OC-rich EVOO induced autophagy through activation of the AMPK/ULK1 pathway. **BBB:** OC-rich EVOO restored BBB function, reducing AD-associated pathology.	[147]
OLE	Experimental spinal cord injury model in rats	The treatment involved administering OLE at a dosage of 20 mg/kg intraperitoneally, immediately and 1 h after the spinal cord injury.	MDA levels were significantly decreased in the OLE treatment groups. GSH levels were significantly increased in the OLE treatment groups. **Apoptosis:** There was a greater Bcl-2 expression and attenuated Bax expression in the OLE-treated rats. TUNEL-positive reactions were significantly reduced in the OLE treatment groups. The treatment with OLE also improved behavioral function compared to the trauma group.	[191]
OLE	TgCRND8 (AD model)	OLE was administered at 50 mg/kg of diet for 8 weeks to 6-month-old TgCRND8 mice.	**PARP1 activation and PAR formation:** OLE treatment rescued PARP1 activation and the levels of its product, PAR, to control values. Neurodegeneration: The beneficial effects of OLE against neurodegeneration were highlighted the TgCRND8 mouse model.	[192]
OLE	5xFAD (AD model)	The administration method involved feeding the mice an OLE-enriched diet at a dosage of 695 mg/kg body weight per day for three months, starting when the mice were three months old.	The study found that OLE reduced neuroinflammation by inhibiting the NF-κB pathway and suppressing the activation of NLRP3 inflammasomes and RAGE/HMGB1 pathways. OLE reduced total Aβ brain levels by increasing clearance and reducing production of Aβ, enhancing BBB integrity and function. These effects collectively improved memory function in the mice. The results suggest that the consumption of OLE as a dietary supplement may stop or slow the progression of AD.	[145]
OleA	Transgenic *Caenorhabditis elegans* strains CL2006 and CL4176, which are simplified models of AD and sporadic inclusion body myositis	The OleA was administered to *Caenorhabditis elegans*, with the CL2006 strain being fed OleA and the CL4176 strain receiving OleA before the induction of the Aβ transgene expression.	**OleA-fed CL2006 worms:** Reduced Aβ plaque deposition. Fewer toxic Aβo. Remarkably decreased paralysis. Increased lifespan compared to untreated animals. **OleA-treated CL4176 worms:** Protective effect observed when OleA was administered before the induction of the Aβ transgene expression. These effects were specific, dose-related, and not mediated by the known polyphenolic antioxidant activity.	[193]
OleA	Rat model	The administration involved injecting a 1.5 µL solution containing the following: OleA (450 µM) or Aggregated Aβ42 peptide (50 µM) in the presence or absence of OleA (450 µM). Control rats were injected with a vehicle (1.5 µL).	Co-administration of OleA counteracted Aβ42 toxicity. The number of choline acetyltransferase-positive neurons in the nucleus basalis magnocellularis was preserved. The number of A11-positive oligomers was lower in the Aβ42-OleA-injected nucleus basalis of Meynert. Glia reaction was lower in the Aβ42-OleA-injected nucleus basalis magnocellularis.	[194]
Olive oil	Mice with induced ischemia-reperfusion	Pre-treatment with olive oil for a week.	Significantly reduced cell death and decreased memory loss.	[195]
Olive oil	Wistar rats	Rats were divided into groups and administered different doses of olive oil (0.25, 0.5, and 0.75 mL/kg/day) by gavage for a specific period.	**Reduction in neurological deficits and infarct volume:** Olive oil administration significantly reduced total neurological deficit scores in rats subjected to middle cerebral artery occlusion. Olive oil at a dose of 0.75 mL/kg/day significantly decreased infarct volume in total, cortex, and striatum areas of the brain. **Molecular effects:** The study measured TNFR1 and NF-κB protein expression in the cortex and striatum regions, indicating involvement in the inflammatory pathway modulation. **Antioxidant and anti-inflammatory effects:** Olive oil contains compounds with these effects, which are beneficial in reducing ischemic damages.	[196]
Olive oil	NAFLD pig model	The administration method involved feeding the pigs diets enriched with olive oil and coconut oil.	The study found that olive- and coconut-oil-enriched diets decreased secondary bile acids. These diets regulated metabolic and transcriptomic markers of brain injury in the frontal cortexes of NAFLD pigs. The results included improvements in various biochemical markers related to inflammation and brain function, suggesting potential neuroprotective effects.	[197]
Olive oil	Mice were injected intracerebroventricularly with Aβ25–35 to induce AD-like pathology	Olive oil was administered orally at a dose of 500 mg/kg/day for 14 days.	**Cognitive function:** Olive oil did not significantly improve performance in the T-maze and novel object recognition tests compared to the control group, indicating limited effect on spatial and recognition memory. In the Morris water maze test, olive oil did not significantly reduce the escape latency or increase the time spent in the target quadrant, suggesting limited improvement in spatial learning and memory. **OS:** Olive oil treatment resulted in a significant decrease in MDA levels in the brain, kidney, and liver, indicating reduced lipid peroxidation. Olive oil reduced NO production in the liver but not significantly in the brain. **Enzyme activity and protein expression:** Olive oil did not significantly inhibit AChE activity in the brain. Olive oil administration slightly reduced the expression levels of iNOS and COX-2, which are markers of inflammation, but the reduction was not as significant as that observed with perilla oil. Olive oil did not significantly upregulate BDNF expression compared to the control group.	[198]
Olive oil	PD induced by rotenone in Swiss albino male adult mice.	Control Group: 2 mL/kg olive oil by oral route. Experimental Groups: 2.5 mg/kg rotenone by intraperitoneal route. Group 3: 7.5 mg/kg Levodopa p.o. Groups 4 and 5: 20 mg/kg and 40 mg/kg embelin p.o., respectively. Groups 6 and 7: 20 mg/kg and 40 mg/kg embelin p.o. respectively, combined with 7.5 mg/kg Levodopa p.o.	The combination of embelin (40 mg/kg) and levodopa (7.5 mg/kg) showed superior neuroprotective activity compared to other treatment groups. This conclusion was based on biochemical parameters, histopathological observations, and immunohistochemical analysis of α-Syn protein in the brain.	[199]
PN	Mice: Cholinergic dysfunction-induced memory impairments, a model for dementia and AD	PN administered at 25 mg/kg orally in a dose-dependent manner.	**Memory improvement:** PN ameliorated memory impairment in the dementia model induced by cholinergic blockade in the passive avoidance test. **Synaptic plasticity:** Facilitated induction of hippocampal long-term potentiation, a cellular model of learning and memory. **Enzyme inhibition**: Blocked AchE activity in a concentration-dependent manner.	[200]
Tyr	*Caenorhabditis elegans* (PD model)	Tyr treatment: Administered to *Caenorhabditis elegans* to study its effects on α-Syn aggregation, neurodegeneration, and OS.	Reduction in α-Syn aggregation: Tyr is effective in reducing α-Syn inclusions. **Decreased toxicity and extended lifespan:** Treated nematodes showed lower toxicity and extended lifespan. **Neuroprotection:** Tyr delayed α-Syn-dependent degeneration of dopaminergic neurons in vivo. **Reduction in OS:** Tyr treatment reduced reactive ROS levels. Promotion of chaperone and antioxidant enzyme expression: Tyr promoted the expression of specific chaperones and antioxidant enzymes.	[201]
VOO. The composition of VOO included fatty acids such as oleic acid (68.822%), linoleic acid (9.951%), palmitic acid (15.99%), stearic acid (1.815%), alpha-linolenic acid (0.602%), and minor components like alpha-tocopherol (210 mg/kg) and total polyphenols (320 mg/kg)	Male Wistar rats (200–300 g)	The treatments administered were: Control group: Rats received saline via gastric gavage. Treatment groups: Rats received 0.25, 0.5, or 0.75 mL/kg/day of VOO via gastric gavage. Treatments were administered daily for 30 days before inducing ischemia-reperfusion injury.	**Reduction in infarct volume:** VOO treatment significantly reduced infarct volumes in the brain compared to controls, with the most significant reduction observed at the highest dose (0.75 mL/kg/day). **Reduction in brain edema:** VOO treatment reduced brain water content in the infarcted hemisphere, indicating reduced brain edema. The doses of 0.5 and 0.75 mL/kg/day were effective, while the lower dose (0.25 mL/kg/day) had no significant effect. **Improvement in BBB integrity:** VOO treatment reduced BBB permeability. The higher doses (0.5 and 0.75 mL/kg/day) significantly reduced BBB permeability compared to the control group. **Improvement in neurological deficit scores:** VOO treatment improved neurological outcomes, with lower neurological deficit scores observed in treated groups compared to controls. **Lipid profile modulation:** VOO treatment reduced the LDL/HDL ratio and increased HDL levels, indicating a favorable effect on blood lipid profiles.	[202]
VOO. The main components of VOO include fatty acids such as oleic acid (C18:1, 76.5%) and linoleic acid (C18:2, 9.9%), along with minor components like alpha-tocopherol (173 mg/kg) and total phenolic compounds (250 mg/kg)	Adult male Wistar rats (200–250 g). Experimental diabetes was induced in the rats using a single femoral intravenous injection of 50 mg/kg streptozotocin	The treatments administered were: - Control group: Normoglycemic rats treated with saline. - Diabetic rats treated with saline. - Diabetic rats treated with 2 mg/kg/day of aspirin orally. - Diabetic rats treated with 0.5 mL/kg/day of VOO orally. - Diabetic rats treated with a combination of aspirin (2 mg/kg/day) and VOO (0.5 mL/kg/day). Treatments were given daily for three months. VOO was administered at 09:00, and aspirin was administered at 17:00 to prevent any pharmacokinetic interactions.	**Neuroprotective effects:** Both VOO and aspirin significantly reduced LDH) efflux after reoxygenation, indicating reduced cell damage (−54.1% for aspirin, −51.3% for VOO, and −72.9% for aspirin plus VOO). **Reduction in lipid peroxides:** Lipid peroxides in brain slices were reduced following treatment with aspirin (−17.9%), VOO (−37.3%), and the combination of both (−49.2%). **Inhibition of nitric oxide production:** The treatments inhibited the production of NO after reoxygenation (−46.5% for aspirin, −48.2% for VOO, and −75.8% for aspirin plus VOO). The activity of inducible iNOS was also reduced (−31.8% for aspirin, −29.1% for VOO, and −56.0% for aspirin plus VOO). **Enhanced antioxidant defense:** The combined treatment of aspirin and VOO resulted in higher GSH levels and increased activities of antioxidant enzymes such as GPx and GSH reductase	[203]

Akt: protein kinase B; Apo-E: Apolipoprotein E; Atf6: activating transcription factor 6; 3NP: 3-nitropropionic acid; Aβ: amyloid beta; AchE: acetylcholinesterase; AD: Alzheimer’s disease; AlCl₃: aluminum chloride; ALS: amyotrophic lateral sclerosis; AMPK: AMP-activated protein kinase; APP: amyloid precursor protein; Bax: Bcl-2-associated X protein; BBB: blood–brain barrier; BDNF: brain-derived neurotrophic factor; Bcl-2: B-cell lymphoma 2; COX-2: cyclooxygenase-2; CREB: cAMP response element-binding protein; DA: dopamine; DOPAC: 3,4-dihydroxyphenylacetic acid; DHA: docosahexaenoic acid; DHPG: 3,4-dihydroxyphenylglycol; ERK: extracellular-related kinase; EVOO: extra virgin olive oil; ER: endoplasmic reticulum; Erβ: estrogen receptor beta FOXO1: forkhead box protein O1; FOXO3: forkhead box protein O3; GAP43: growth-associated protein 43; GCLC: glutamate–cysteine ligase catalytic subunit; GCLM: glutamate–cysteine ligase modifier subunit; GFAP: glial fibrillary acidic protein; GLUT1: glucose transporter 1; GPx: glutathione peroxidase; Grp78: glucose-regulated protein 78; GSH: glutathione; HDL: high-density lipoprotein; HMGB1: high mobility group box 1 protein; HO-1: heme oxygenase-1; Hsp27: heat shock protein 27; HT: hydroxytyrosol; HT-AC: hydroxytyrosol acetate; HTEE: hydroxytyrosol ethyl ether; iNOS: inducible nitric oxide synthase; JAK2: janus kinase 2; JNK: Jun N-terminal kinase; LDH: lactate dehydrogenase; LDL: low-density lipoprotein; LRP1: low density lipoprotein receptor-related protein 1; MAPK: mitogen-activated protein kinase; MDA: malondialdehyde; MHTEE: 3,4-di-O-methylidene-hydroxytyrosol ethyl ether; MPTP: 1-methyl-4-phenyl-1,2,3,6-tetrahydropyridine; NAFLD: non-alcoholic fatty liver disease; NGF: nerve growth factor; NLRP3: NOD-like receptor pyrin domain containing 3; NMDAR1: N-methyl-D-aspartate receptor 1; NMDANR2A: N-methyl-D-aspartate receptor subunit 2A; NMDANR2B: N-methyl-D-aspartate receptor subunit 2B; 3NP: 3-nitropropionic acid; NO: nitric oxide; NF-κB: nuclear factor kappa-light-chain-enhancer of activated B cells; Nrf2: nuclear factor erythroid 2-related factor 2; OA: oleic acid; OLC: oleocanthal; OLE: oleuropein; OleA: oleuropein aglycone; OOLF: lipophilic fraction of olive oil; OOHF: hydrophilic fraction of olive oil; OS: oxidative stress; PAR: poly(ADP-ribose); PARP1: poly(ADP-ribose) polymerase 1; PD: Parkinson’s disease; P-gp: P-glycoprotein; PGE2: prostaglandin E2; PGC-1α: peroxisome proliferator-activated receptor gamma coactivator 1-alpha; PI3K: phosphoinositide 3-kinase; PN: (+)-pinoresinol; PS1: presenilin1; PSD-95: postsynaptic density protein 95; RAGE: receptor for advanced glycation end products; ROS: reactive oxygen species; RSK2: ribosomal S6 kinase 2; SIRT1: sirtuin 1; SNAP-25: synaptosomal-associated protein of 25 kDa; SOD1: superoxide dismutase 1; TEE: tyrosol ethyl ether; TUNEL: terminal deoxynucleotidyl transferase dUTP nick-end labelling; Tyr, Tyrosol; VOO: virgin olive oil; α-Syn: alpha-synuclein.

**Table 5:** Clinical trials of olive oil effects in cognitive function.

Status	Study	Key Findings/Aim	Reference
Complete	Three-city study	Olive oil consumption associated with lower odds of cognitive deficits and decline.	[204]
	PREDIMED-NAVARRA	Mediterranean diet with EVOO or nuts improves global cognition independently of potential confounders.	[205]
	MICOIL Pilot Study	High and moderate phenolic EVOO linked to improved cognitive performance, potentially preventing MCI progression to Alzheimer’s.	[206]
	AU-ROOAD	EVOO reduces blood–brain barrier permeability, lowers amyloid-B levels, and improves dementia symptoms.	[207]
Ongoing	GOLDENNCT04440020	Investigates the effects of a Mediterranean diet and olive leaf beverages on memory and cognitive function.	[208]
	NCT05363267	Studies the combined effects of high-phenolic EVOO and curcumin on neurofibromatosis, Type 1.	[209]
	NCT05929924	Assess whether EVOO has a protective effect against AD in healthy individuals whose family history includes the disease.	[210]

AD: Alzheimer’s disease; EVOO: extra virgin olive oil; MCI: mild cognitive impairment.
